# Supplementary material for: Undernutrition and its determinants among adolescent girls in low land area of Southern Ethiopia
Source: PLoS One. 2021 Jan 12;16(1):e0240677. doi: 10.1371/journal.pone.0240677 (PMC7802945; doi:10.1371/journal.pone.0240677)
Supplement: S1 Checklist — (DOCX) [file pone.0240677.s001.docx]

**STROBE Statement**

Checklist of items that should be included in reports of observational studies

| **Section/Topic** | Item No | Recommendation | Reported on Page No |
| --- | --- | --- | --- |
| **Title and abstract** |  | (*a*) **Undernutrition and its determinants among adolescent girls in low land area of southern Ethiopia: Community based cross-sectional study** | 1 |
|  |  | (*b*) *Undernutrition is one of the most common causes of morbidity and mortality among adolescent girls worldwide, especially in South-East Asia and Africa. The objective of this study was to assess the nutritional status and associated factors among adolescent girls in the Wolaita and Hadiya zones* of *Southern Ethiopia. A community-based cross-sectional study was conducted, and a multistage random sampling method was used to select a sample of 843 adolescent girls. Anthropometric measurements were collected from all participants and entered in the WHO Anthro plus software for Z-score analysis. The data was analyzed using EPI-data 4.4.2 and SPSS version 21.0. The odds ratios for logistic regression along with a 95% confidence interval (CI) were* generated*. A P- value <0.05 was declared as the level of statistical significance. Thinness (27.5%) and stunting (8.8%) are found to be public health problems in the study area. Age, large family size, low monthly income, not taking deworming tablets, low educational status of the father, the source of food for the family only from market, not visited by health extension workers, and not washing hand with soap before eating and after using the toilet were positively associated with poor nutritional status of adolescent girls in the Wolaita and Hadiya zones, Southern Ethiopia.* | 2 |
| Introduction | | | |
| Background/rationale | 2 | Adolescence represents a window of opportunity to prepare nutritionally healthy adult life. Some nutritional problems originating early in life can be potentially corrected, in addition to addressing and maintaining the nutritional status. It may also be timely period to shape and consolidate healthy eating and lifestyle behaviors, thereby preventing or postponing the onset of nutrition-related chronic disease in adulthood. A good understanding of nutritional status of adolescent girls will have many important public health and policy implications. | 2 |
| Objectives | 3 | 1. To determine prevalence of thinness and stunting among adolescent girls in Southern Ethiopia  2. To identify determinants of thinness and stunting among adolescent girls in Southern Ethiopia | 2 |
| Methods | | | |
| Study design | 4 | A community-based cross-sectional study was conducted | 5 |
| Setting | 5 | The study was conducted in the Wolaita and Hadiya zones of Southern Ethiopia. These zones are predominantly dependent on agriculture, practicing mixed crop-livestock production and living in permanent settlements. Data was collected from April 30, 2019 to May 30, 2019. | 5 |
| Participants | 6 | ***Cross-sectional study*** — Adolescent girls age range within 10-19years who are physically and mentally normal/not ill. |  |
| Variables | 7 | **Outcomes variables -** BMI-for-age Body mass index for age z-score and height-for-age z-score were the dependent variables  **Predictors variables**: Age, educational status of the participant, family size, maternal and paternal educational level, access to nutritional counselling services in health facilities, deworming tablets, iron-folic acid supple­mentation, household monthly income, source of food, and number of meals per day. | 5 |
| Data sources/measurement | 8* | *Data sources:* Anthropometrics (height and weight) were measured on all sampled adolescent girls. Weight was measured to the nearest 100 g using a standard SECA digital scale while the participants wore light clothing and no shoes. The scale was calibrated after weighing each participant. Height was measured in a standing position to the nearest 0.1 cm using a vertical board with a detachable sliding headpiece. Measuring tape was attached to it. BMI-for- age z-scores and height-for-age z-scores were calculated. In addition to this socioeconomic and demographic data were collected by using structured interviewer-administered questionnaire. | 6-7 |
| Bias | 9 | -The questionnaire was prepared in English and then translated to Amharic and rendered back to English to keep the consistency of the questions.  - Data collectors and supervisors were trained for 4 days to properly fill out the questionnaire and measure anthropometry.  -Data collectors were selected from each zone so they could communicate fluently in the local language and understand the socio-cultural practices of the community.  -The questionnaire was pre-tested on 5% adolescent girls in a similar area to the study sites to ensure reliability.  - Anthropometric measurements were standardized .Technical error of measurement and coefficient of variance (CV) were computed for all data collectors by using ENA for SMART software. Data collators with unacceptable TEM and CV were allowed to repeat the steps again. | 7-8 |
| Study size | 10 | A single population proportion formula, [n=z$\frac{\propto}{2}$^2^P (1-P) /d^2^] was used to estimate the sample size. From the literature review, the prevalence of thinness (24.4%) and stunting (29.4%) were used for sample size calculations. Sample size calculation by using thinness (24.4%) was n= (Z _α/2_)^2^*p (1-p)/d^2^ = 748 and sample size calculation by using stunting (29.4%) was n= n= (Z _α/2_)^2^*p (1-p)/d^2^ = 843**.** So that for this study, stunting (29.4%) was selected to estimate the sample size as it gives a larger sample; considering a 95% confidence interval (CI) and d=0.05%, the initial sample size was 383. By adding 10% for non-response and a design effect of 2.4, the final sample size was **843**. n= (Z _α/2_)^2^*p (1-p) DE /d^2^_._ Where: Z = Standard normal distribution value at 95% CI = (1.96)^2^, DE **=** design effect, and d = 0.05 (5% margin of error). | 5-6 |
| Quantitative variables | 11 | First, the data was checked for completeness and consistency for data entry and cleaning. Then, data was entered in the computer using EPI-data version 4.4.2 and exported to SPSS version 21.0 for further analysis. | 7 |
| Statistical methods | 12 | Descriptive statistics such as frequencies, proportions, and cross-tabulation were used to present the data. In addition, bivariate logistic regression analysis was performed to assess the association between independent and dependent variables. Variables that showed an association (p-value ≤ 0.25) in the bivariate analysis were included in the final multivariate logistic regression model. Odds ratios for logistic regression along with a 95% CI were estimated. A p-value less than 0.05 was declared statistically significant. | 7-8 |

| Participants | 13* | For this study 843 adolescent girls planned to be included in the study. But only 820 adolescent girls were participated in the study. From the total 23 adolescent girls were refused to participate. | 9 |
| --- | --- | --- | --- |
| Descriptive data | 14 | The mean age of the study participants was 14.6 (±1.9) years, the mean family size was 6.56 (±1.83) persons, while 69.3% of the households had ≥ 5 family members and 30.7% had < 5 family members. Most of the study participants (93.3%) were in grades 1-8 and only 0.2% had college and University education. Most of the study participants were Protestant (65.0%), but 34.3% were Orthodox Christian, and only 0.7% were Muslims. About one third (33.4%) of the study participants were from households that have less than 1000 ETBirr(31.25 USD) monthly income and 30.3% are from households that have greater than 2000 ETBirr (62.5USD) monthly income. | 9 |
|  |  | No participants with missing data in our study. |  |
|  |  | *Cross-sectional study—*Report numbers of outcome events or summary measures  **Response:** The mean age of the study participants was 14.6 (±1.9) years, the mean family size was 6.56 (±1.83) persons, while 69.3% of the households had ≥ 5 family members and 30.7% had < 5 family members. Most of the study participants (93.3%) were in grades 1-8 and only 0.2% had college and University education. |  |
| Main results | 16 | *Thinness (27.5%) and stunting (8.8%) are found to be public health problems in the study area. Age [AOR(adjusted odds ratio) (95% CI) = 2.91 (2.03-4.173)], large family size [AOR (95% CI) = 1.63(1.105-2.396)], low monthly income [AOR (95% CI) = 2.54(1.66-3.87)], not taking deworming tablets [AOR (95% CI) = 1.56(1.11-21)], low educational status of the father [AOR (95% CI) = 2.45(1.02-5.86)], the source of food for the family only from market [AOR (95% CI) = 5.14(2.1-12.8)], not visited by health extension workers [AOR (95% CI) = 1.72(1.7-2.4)], and not washing hand with soap before eating and after using the toilet [AOR (95% CI) = 2.25(1.079-4.675)] were positively associated with poor nutritional status of adolescent girls* | 15-18 |
| Other analyses | 17 | No other analyses were done. | NA |
| Discussion | | | |
| Key results | 18 | -19.5% of the adolescent girls were moderately thin and 8% were severely thin.  - 7.8% of the adolescent girls were moderately stunted and 1% are severely stunted. | 18 |
| Limitations | 19 | Using cross-sectional study design might have chicken egg dilemma for some variables in the study | 23 |
| Interpretation | 20 | Thinness and stunting were found to be high in the study area. Age, family size, monthly household income, fathers’ educational status, visits by health extension workers, and nutrition services decision-making power are the main predictors of thinness. Hand washing practice, visits by health extension workers, and nutrition services decision-making power are the main predictors of stunting among adolescent girls in Southern Ethiopia. | 22 |
| Generalisability | 21 | Thinness and stunting were found to be high in the study area. At all levels, the girls' nutrition education /counselling should be given due emphasis as they are tomorrow's mothers which is very important to break the intergenerational cycle of malnutrition. | 22 |
| Other Information | | | |
| Funding | 22 | Addis Ababa University center for food science and nutrition and Wolaita Sodo University. Additionally, small research grant from Tufts University. | 24 |

**Give information separately for cases and controls in case-control studies and, if applicable, for exposed and unexposed groups in cohort and cross-sectional studies.*

**Note:** An Explanation and Elaboration article discusses each checklist item and gives methodological background and published examples of transparent reporting. The STROBE checklist is best used in conjunction with this article (freely available on the Web sites of PLoS Medicine at http://www.plosmedicine.org/, Annals of Internal Medicine at http://www.annals.org/, and Epidemiology at http://www.epidem.com/). Information on the STROBE Initiative is available at www.strobe-statement.org.
