# Supplementary material for: Undernutrition and its determinants among adolescent girls in low land area of Southern Ethiopia
Source: PLoS One. 2021 Jan 12;16(1):e0240677. doi: 10.1371/journal.pone.0240677 (PMC7802945; doi:10.1371/journal.pone.0240677)
Supplement: S1 File — (DOCX) [file pone.0240677.s002.docx]

Written information sheet, assent and consent form

| **Introduction and Ascent/Consent** |
| --- |
| **Introduction/Purpose:**  My name is___________ and I am working as data collector for PhD student Yoseph Halala. He is student at Center for Food Science and Nutrition, Addis Ababa University. He is conducting an assessment on nutritional and health behaviors of adolescent girls (10-19 years) with the financial support of Tufts university.  **Procedures:**  This information will help us to plan interventions that improve nutrition service seeking behavior among adolescent girls. The questionnaire usually takes about 45 minutes to complete. Additionally, I would like to take some weight, height and MUAC measurements of the participants. This information will allow us to assess the health and nutritional status of adolescents in the study area.  **Risks:**  I believe there are no risks to adolescent girl from participating in this study. The adolescent girls should not experience any discomfort whatsoever.  **Benefits:**  Taking part in this research study may not benefit participants personally, but it may help us to improve future nutrition interventions to improve the nutritional and health status of adolescents in the study areas.  **Compensation**  Participants will not be paid for participating in this study. I appreciate their participation in this study.  **Confidentiality:**  We will keep participants’ answers confidential to the best of our ability. We will not share any answers with any of your neighbors or family. We will use a number instead of name on study forms. The name and other facts that might help people recognize individual will not appear when we present this study to others or publish its results. Any information that might identify participant will be kept separate from the answers, and the answers will be kept in a secure place for analyses by the researchers only. The original paper questionnaires will be kept in locked cabinets for 3 years and the data must be stored on password-protected computers.  **Voluntary Participation and Withdrawal:**  Taking part in this study is voluntary. Participant can choose not to talk to us or ask us to leave, and if Participant does agree to participate, they can stop the interview at any time or skip any questions that they don’t want to answer. There is no right or wrong answer. We just want to hear more about their own ideas and experiences.Whether or not they choose to participate will not affect the services that they may receive from the health system in any way.  **Contact Persons:**  If you have any questions, I will be happy to answer them. And in case you ever want to contact someone from the office we work for, I can write down the telephone number for the Principal Investigator from Addis Ababa University. You should call this number and ask for Yoseph Halala: 0913233179.  Do you have any questions about the survey? Do you have any questions about the measurements we will take of you/your adolescent girl? Please let me know if anything I have stated is not clear and I will be happy to explain it further to ensure you understand.  **Consent forms for adolescent girls [age 18- 19 years]**  I (-----------------------------------) have been informed about the study entitled “Assessing barriers and facilitators of nutrition service utilization and associated factors among adolescent girls in Southern Ethiopia  will be conducted by Mr. Yoseph Halala.  I understand the purpose and procedures of the study. I have been given an opportunity to answer questions about the study and have had answers to my satisfaction.  I declare that my participation in this study is entirely voluntary and I can withdraw at any time without affecting any treatment or care that I would usually be entitled to.  I have been informed about any available compensation or medical treatment if injury occurs to me as a result of study-related procedures.  We appreciate your participation in this study. I hope that you participate, since your opinion is important.  If you are willing to participate, please put your signature or thumb print in the space provided below.  Participant's signature or left thumb print: ____________________ Date: ______________  Interviewer's signature: ____________________ Date: ______________  Thank you for your cooperation.  **Consent form for parent/guardian of adolescent girls [age 10-18 years)**  I (-----------------------------------) have been informed about the study entitled “Assessing barriers and facilitators of nutrition service utilization and associated factors among adolescent girls in Southern  Ethiopia” will be conducted by Mr. Yoseph Halala.  I understand the purpose and procedures of the study. I and my adolescent girl have been given an opportunity to answer questions about the study and have had answers to her satisfaction. I declare that her participation in this study is entirely voluntary and she can withdraw at any time without affecting any treatment or care that she would usually be entitled to.  I have been informed about any available compensation or medical treatment if injury occurs to my girl as a result of study-related procedures.  We appreciate your willingness and her participation in this study. I hope that you will give permission to your adolescent girl to participate, since her opinion is important.  If you have agreed the participation of your girl in this study, please put your signature or thumb print in the space provided below.  Parent/guardian’s signature or left thumb print: ____________________ Date: ____________  Interviewer's signature: ____________________ Date: ______________  Thank you for your cooperation.  **Assent forms for adolescent girls [age 10- 18 years)**  Hello, my name is ___________and I am working as data collector of Yoseph Halala for PhD study.  **Protocol** **Title:** Assessing barriers and facilitators of nutrition service utilization and associated factors among adolescent girls in Southern Ethiopia. I would very much appreciate your participation in this study. This information will help the government to plan health and nutrition services. Among all adolescent girls in this zone, you have been chosen randomly to participate in this study, and your parents/ guardian have said that it is okay and I have got full permission from your parent/ legal guardians. The study will take about 45 minutes of your time. If you have any questions after the study is over, talk to your parents. Do you have any questions now about being in the study?  I understand the purpose and procedures of the study. I have been given an opportunity to answer questions about the study and have had answers to my satisfaction.  I declare that my participation in this study is entirely voluntary and I can withdraw at any time without affecting any treatment or care that I would usually be entitled to.  I have been informed about any available compensation or medical treatment if injury occurs to me as a result of study-related procedures.  We appreciate your participation in this study. I hope that you participate, since your opinion is important.  If you are willing to participate, please put your signature or thumb print in the space provided below.  Participant's signature or left thumb print: ____________________ Date: ______________  Interviewer's signature: ____________________ Date: ______________  Thank you for your cooperation. |

## Annex II. An interview Questionnaire

| Code | Question | Response | | Skip |
| --- | --- | --- | --- | --- |
|  | **Part 1. Survey information** |  | |  |
| 101 | Name of Interviewer | __________________ | |  |
| 102 | Participant’s ID number | __________________ | |  |
| 103 | Date of data collection | _____/_____/­­­_____ | |  |
| 104 | Address | Zone________Woreda__________  Kebele ______________ | |  |
|  | **Part 2. Socio-demographic information** |  | |  |
| 201 | What is your date of birth? dd/mm/ yy | _______ | |  |
| 202 | How old are you? (In completed years) | ______ | |  |
| 203 | What is your religion? | Orthodox -----------------------------------01  Protestant ----------------------------------02  Muslim ------------------------------------03  Other specify ------------------------------99 | |  |
| 204 | Are you in school or out of school? | In school --------------------------------------01  out of school ----------------------------------02 | |  |
| 205 | What is the highest level of education you have completed? | No formal education ---------------01  1-4 grade ----------------------------02  5-8 grade ----------------------------03  9-10 grade --------------------------04  11-12 grade-------------------------05  College and University------------06 | |  |
| 206 | Family size residing in the your home | [__][__] (write number) | |  |
| 207 | Occupation of father/household head? | Farmer/ Agricultural worker ----------01  Skilled laborer --------------------------02 Unskilled laborer -----------------------03  Business/ Traders-----------------------04 Professional jobs -----------------------05  Work at home---------------------------06 Jobless -----------------------------------07 Other (Specify)------------------------------99 | |  |
| 208 | Occupation of mother | Farmer/ Agricultural worker ----------01  Skilled laborer --------------------------02 Unskilled laborer -----------------------03  Business/ Traders-----------------------04 Professional jobs -----------------------05  Work at home---------------------------06 Jobless -----------------------------------07 Other (Specify)-------------------------------99 | |  |
| 209 | The educational status of your father | No formal education ---------------01  1-4 grade ----------------------------02  5-8 grade ----------------------------03  9-10 grade --------------------------04  11-12 grade-------------------------05  College and University------------06 | |  |
| 210 | The educational status of your mother | No formal education ---------------01  1-4 grade ----------------------------02  5-8 grade ----------------------------03  9-10 grade --------------------------04  11-12 grade-------------------------05  College and University------------06 | |  |
| 211 | What is the average monthly income of your family (ETB)?  (Ask their parents/guardians for this question) | _______________________ | |  |
|  | **Part 3: Health institution and nutrition service related questions** | | |  |
| 301 | Is there Accessible  Health post ? | | Yes ---------------01  No ---------------02 |  |
| 302 | Is there Accessible Health center access? | | Yes ---------------01  No ---------------02 |  |
| 303 | Is there Accessible  Hospital access? | | Yes ---------------01  No ---------------02 |  |
| 304 | What is the distance between your home and the nearest health facilities? | | --------------------- Km  I don’t know -------88 |  |
| 305 | How long does it take to go the nearest health institution? (single trip) | | \|___\|____\|____\| , Minutes |  |
| 306 | What is means of transportation to nearest health institution? | | Walking --------------------------01  Vehicle/public transport -------02  Mules/horse ----------------------03  I don’t know -------88 |  |
| 307 | Do you have any information about the benefit of having health institution nearby? | | Yes -----------------01  No -----------------02 |  |
| 308 | If yes for Q307, what is the primary source of information? | | Health workers--------01  Friends/ neighbors----02  Radio, TV -------------03  Parents/ Guardians ---04  Other specify---------------------------------99 |  |
| 309 | Who is decision-maker in your family for you to get nutrition service? | | Father -----------------------------------01  Mother -----------------------------------02  Jointly (both mother & father ) -------03  Other (specify) ----------------------------99 |  |
| 310 | Have you ever taken nutrition counseling/education services in the last six months? | | Yes -----------------01  No -----------------02 |  |
| 311 | If yes for Q310, Who have given nutrition counseling /education services for you? | | Health extension workers------------01 Nurses/ health officer ----------------02  Doctors ---------------------------------03  Other (specify) ----------------------------99 |  |
| 312 | If yes for Q310, Where have you taken nutrition counseling/education services? (multiple answers possible) | | Health facility(HC, HP & Hospital)---01  School ---------------------------------02  Home/community through health extension--------------------------03  Other (specify)____________________99 |  |
| 313 | Have you received any deworming tablet in the past 6 months? | | Yes -----------------01  No -----------------02 |  |
| 314 | If yes for Q313, how many deworming tablet per six months? | | --------------------specify the number |  |
| 315 | If yes for Q314, from where did you receive the deworming? | | Health facility(HC, HP & Hospital)---01  School -------------------------------------02  Home/community through health extension--------------------------03  Other (specify)____________________99 |  |
| 316 | Have you received any iron-folic acid tablets in the past 6 months? | | Yes -----------------01  No -----------------02 |  |
| 317 | If yes for Q316, how (from where) did you receive the iron-folic acid supplements? | | Health facility(HC, HP & Hospital)---01  School -------------------------------------02  Home/community through health extension--------------------------03  Other (specify)___________________99 |  |
| 318 | Do you think that healthcare providers are providing friendly nutrition service from the perspective of quality?  (are they giving complete and clear information for you?) | | Yes -----------------01  No -----------------02 |  |
| 319 | Have you been ill with a cough or breathing problems in the past 2 weeks? | | Yes -----------------01  No -----------------02 |  |
| 320 | Have you been diagnosed with anemia in the past six months? | | Yes -----------------01  No -----------------02 |  |
| 321 | Have you been ill with diarrhea in the past 2 weeks (3 or more loose or watery stools in a 24-hour period)? | | Yes -----------------01  No -----------------02 |  |
| 322 | Have you been ill with malaria in the past 2 weeks? | | Yes -----------------01  No -----------------02 |  |

## Part IV: Behavior and lifestyle of adolescents

| 401 | | Do you smoke cigarette? | | Yes -----------------01  No -----------------02 | If no skip to Q403 | |
| --- | --- | --- | --- | --- | --- | --- |
| 402 | | If yes for Q401, how old were you when you first tried cigarette smoking? | | ------------------------years |  | |
| 403 | | Have you ever drink alcohol? | | Yes -----------------01  No ------------------02 | If no skip to Q405 | |
| 404 | | During the past 30 days on how many days did you have at least one drinking containing alcohol? | | One -------------------01  Two-------------------02  Three and above----03 |  | |
| 405 | | Do you chew khat? | | Yes -----------------01  No -----------------02 | If no skip to Q501 | |
| 406 | | If yes, how old were you when you first tried chewing khat? | | ---------------------years |  | |
| **Part V: Personal hygiene and environmental-sanitation information** | | | | | | |
| 501 | Do your family have latrine? | | Yes -----------------01  No -----------------02 | | | If no skip to Q503 |
| 502 | What kind of toilet facility do members of your household usually use? | | **Flush or pour flush toilet**  Flush to piped sewer system . . .. . 01  Flush to septic tank………... . . . . 02  Flush to pit latrine..……. . . . . . . 03  Flush to somewhere else . . . . . . . 04  Flush, don't know where ...….. . . 05  **Pit latrine**  Ventilated improved pit latrine . . 06  Pit latrine with slab . ....……... . . 07  Pit latrine without slab/open pit . . 08  Composting toilet . . . . . . ..… . . . 09  Bucket toilet . . . . . . . ……. . . . . 10  Hanging toilet/hanging latrine . .. 11  No facility/bush/field . . ... ….. . ..12  Other ---------------------------------99 | | |  |
| 503 | What is the main source of drinking water for members of your household? | | **Piped water**  Piped into dwelling . . ... . . . . 01  Piped to yard/plot . . . . . . . .. .02  Piped to neighbor . . . . . . .. . .03  Public tap/standpipe . . . . . . .04  Tube well or borehole . . . . . 05  **Dug well**  Protected well . . . . . . . . . . . . 06  Unprotected well . . . . .. . . . . 07  **Water from spring**  Protected spring . . . . . . . . . . 08  Unprotected spring . . . . . .. . 09  Rainwater . . . . . . . . . . . . . . . 10  Tanker truck (boti) . . .. . . . . . 11  Cart with small tank . . …... . 12  **Surface water** (river/dam/  Lake/pond/stream/canal/  Irrigation channel) . . . … . . . 13  Bottled water . . . . . . .. . . . . . 14  Other …………….……………..99 | | |  |
| 504 | How long does it take to collect water (single trip)? | | Less than five minute ---------01  5-15 minutes -------------------02  15-30 minutes------------------03  30 minutes – 1 hour-----------04  Greater than 1 hour------------05 | | |  |
| 505 | How many people slept in your house last night? | | -------------(enter number) | | |  |
| 506 | Do your animals live in the same house where you are living? | | Yes -----------------01  No -----------------02 | | |  |
| 507 | Housing condition: number of rooms | | _______________________ | | |  |
| 508 | Number of windows you are living | | _______________________ | | |  |
| 509 | Type of floor you are living | | Cement----------------01  Muddy ----------------02  Other specify________________99 | | |  |
| 510 | How many times per day did you usually clean or brush your teeth? | | ------------( enter number) | | |  |
| 511 | How often did you wash your hands before eating? | | Not at all ----------------01  Sometimes---------------02  Ussually -----------------03 | | |  |
| 512 | Do you wash your hands after using the toilet or latrine? | | Yes -----------------01  No -----------------02 | | |  |
| 513 | How often did you wash your hands after using the toilet or latrine? | | Not at all ----------------01  Sometimes---------------02  Ussually -----------------03 | | |  |
| 514 | Do you use soap when washing your hands? | | Yes -----------------01  No -----------------02 | | | If no skip to Q 601 |
| 515 | If yes for Q514, how often do you use soap when washing your hands? | | Not at all ----------------01  Sometimes---------------02  Ussually -----------------03 | | |  |

**Part VI. Nutrition and dietary information of adolescent’s girls**

| 601 | What is your staple food? ( more than one answer is possible) | Teff ------------------------01  Maize------------------------02  Sorgume---------------------03  Barley-----------------------04  wheate ----------------------05  Enset ------------------------06  Other specify--------------------99 |  |
| --- | --- | --- | --- |
| 602 | Where do you get food for family or personal consumption? | Own product-------------------01  Market purchase---------------02  Own product and market purchase------------------------03  Food aid -----------------------04  Gift -----------------------------05  Food loan----------------------06  Other specify--------------------99 |  |
| 603 | Usually, how many meals per day are you getting? | One time ------------------------01  Two times------------------------02  Three times----------------------03  Four times and above ----------04 |  |
| 604 | Are you skipping your regular meal? | Yes -----------------01  No ------------------02 |  |
| 605 | If yes for Q604, which meal do you skip usually? | Breakfast -------------01  Lunch------------------02  Dinner ----------------03  Snack------------------04 |  |
| 606 | Are you consuming coffee and tea with or after meal? | After meal----01  With meal-----02 |  |
| 607 | If answer after meal for Q 606, after how many minute after meal? | --------------------------------- |  |

**Part 7: Food Security**

**For each of the following questions, ask the adolescent girl/her parents to consider what has happened in the past 30 days**

| 701 | In the past 30 days did you worry that your household would not have enough food? | Yes--------------- 1  No --------------2 | If answer is “No” skip to >702 |
| --- | --- | --- | --- |
| 701_1a | If "Yes", how often did this happen? | Rarely (1-2 times)----------- 01  Sometimes (3-10 times)- -----02  Often (more than 10 times) -----03 |  |
| 702 | In the past 30 days were you or any household members not able to eat the kinds of foods you preferred because of a lack of resources? | Yes -----------------1  No- -----------------2 | If answer is “No” skip to >703 |
| 702_a | If "Yes", how often did this happen? | Rarely (1-2 times)----------- 01  Sometimes (3-10 times)- -----02  Often (more than 10 times) -----03 |  |
| 703 | In the past 30 days did you or any household member eat just a few kinds of food day after day because of a lack of resources? | Yes -----------------1  No- -----------------2 | If answer is “No” skip to >704 |
| 703_a | If "Yes", how often did this happen? | Rarely (1-2 times)----------- 01  Sometimes (3-10 times)- -----02  Often (more than 10 times) -----03 |  |
| 704 | In the past 30 days did you or any household member eat food that you did not want to eat because of a lack of resources to obtain other types of food? | Yes -----------------1  No- -----------------2 | If answer is “No” skip to >705 |
| 704_a | If "Yes", how often did this happen? | Rarely (1-2 times)----------- 01  Sometimes (3-10 times)- -----02  Often (more than 10 times) -----03 |  |
| 705 | In the past 30 days did you or any household member eat a smaller meal than you felt you needed because there was not enough food? | Yes -----------------1  No- -----------------2 | If answer is “No” skip to >706 |
| 705_a | If "Yes", how often did this happen? | Rarely (1-2 times)----------- 01  Sometimes (3-10 times)- -----02  Often (more than 10 times) -----03 |  |
| 706 | In the past 30 days did you or any household member eat fewer meals in a day because there was not enough food? | Yes -----------------1  No- -----------------2 | If answer is “No” skip to >707 |
| 706_a | If "Yes", how often did this happen? | Rarely (1-2 times)----------- 01  Sometimes (3-10 times)- -----02  Often (more than 10 times) -----03 |  |
| 707 | In the past 30 days was there ever no food at all in your household because there were no resources to get more? | Yes -----------------1  No- -----------------2 | If answer is “No” skip to >708 |
| 707_a | If "Yes", how often did this happen? | Rarely (1-2 times)----------- 01  Sometimes (3-10 times)- -----02  Often (more than 10 times) -----03 |  |
| 708 | In the past 30 days did you or any household member go to sleep at night hungry because there was no enough food? | Yes -----------------1  No- -----------------2 | If answer is “No” skip to >709 |
| 708_a | If "Yes", how often did this happen? | Rarely (1-2 times)----------- 01  Sometimes (3-10 times)- -----02  Often (more than 10 times) -----03 |  |
| 709 | In the past 30 days did you or any household member go a whole day without eating anything because there was no enough food? | Yes -----------------1  No- -----------------2 | If answer is “No” skip to >710 |
| 709_a | If "Yes", how often did this happen? | Rarely (1-2 times)----------- 01  Sometimes (3-10 times)- -----02  Often (more than 10 times) -----03 |  |

**Part 8. Nutrition Education**

| **S.N.** | **Questions** | | | **Choices/Answers** | | **Skip** |  |  |
| --- | --- | --- | --- | --- | --- | --- | --- | --- |
| 801 | Have you been visited by a HEW at your home any time in the last three months? | | | Yes ---------1  No ----------2  I Can’t remember----88 | | >806  >806 |  |  |
| 802 | How many times did a HEW visit you at your home in the last three months? | | | One time ------- 01  Two times ---------02  Three times ---------03  Four or more times ------ 04  Do not remember -------88 | |  |  |  |
| 803 | When was the last time that a HEW visited you at home? | | | Within last 1 month -------01  1-3 months ago -------02  Do not remember/know -------88 | |  |  |  |
| 804 | The last time when a HEW visit you at home, did she speak with you about adolescent nutrition? | | | Yes --------1  No ---------2 | | >806 |  |  |
| 805 | Could you tell me what the HEW told you about adolescent nutrition?  (Probe deep to find out more about this information . Multiple responses allowed) | | | Eating a diversified diet ---------01  Taking iron-folic acid supplement-02 Taking deworming tablet -----03  Other (specify) -------99  __________________________ | |  |  |  |
| 806 | Did you have a contact with an HEW in the community (any time in the last three months?) | | | Yes --------------1  No ---------------2  I Can’t remember-88 | | >811  >811 |  |  |
| 807 | How many times did you have a contact with an HEW in the community outside your home or health post in last three months? | | | One time ------- 01  Two times ---------02  Three times ---------03  Four or more times ------ 04  Do not remember -------88 | |  |  |  |
| 808 | The last time you had a contact with an HEW in the community, where did this contact occur? | | | At a village gathering----------01  During PSNP Pay day----------02  At religion place-----------03  During outreach services------------04  other specify ---------------99  ____________________________ | |  |  |  |
| 809 | The last time when you had a contact with an HEW in the community, did she speak with you about adolescent nutrition? | | | Yes ---------------------1  No --------------------2 | | >1211 |  |  |
| 810 | During your last contact with an HEW in the community, what did the HEW tell you about adolescent nutrition? (*Do not read out the options provided. Probe deep to find out more about the information, multiple responses possible)* | | | Eating a diversified diet ---01  Taking iron-folic acid supplement -02 Taking deworming tablet --03  Other (specify) --99  ___________________________ | |  |  |  |
| 811 | Have you been visited by an HDA or WDA at your home any time in the last three months? | | | Yes -----------1  No ------------2  I Can’t remember------88 | | >1216  >1216 |  |  |
| 812 | How many times did an HDA or WDA visit you at your home in the last three months? | | | One time ------- 01  Two times ---------02  Three times ---------03  Four or more times ------ 04  Do not remember -------88 | |  |  |  |
| 813 | When was the last time that an HDA or WDA visited you at home? | | | Within last 1 month -------01  1-3 months ago--------02  3-6 months ago -------03  Do not remember/know ------88 | |  |  |  |
| 814 | The last time when an HDA/WDA visit you at home, did she speak with you about adolescent nutrition? | | | Yes -------------1  No --------------2 | | >1216 |  |  |
| 815 | Could you tell me what the HDA/WDA told you about adolescent nutrition at your home?(multiple responses allowed) | | | Eating a diversified diet ----01  Taking iron-folic acid supplement --2 Taking deworming tablet ---03  Other (specify) ------99  ____________________________ | |  |  |  |
| 816 | In the past 6 months, have you attended an event at in the community that was related to adolescent nutrition? | | | Yes --------1  No --------2 | |  |  |  |
| 817 | Have you had nutrition education sessions in your school in the last three months? | | | Yes -------1  No -------2  I Can’t remember----88 | | >900  >900 |  |  |
| 818 | How many times did you have nutrition education in the last three months? | | | One time ------- 01  Two times ---------02  Three times ---------03  Four or more times ------ 04  Do not remember -------88 | |  |  |  |
| 819 | What was the nutrition education about? | | | Eating a diversified diet ---01  Taking iron-folic acid supplement -02 Taking deworming tablet ---03  Other (specify) ----99  _____________________________ | |  |  |  |
| Part 9: Mass/multi-media coverage | | | | | | | |  |
| **S.N.** | | **Questions** | **Choices/Answers** | | **Skip** | | |  |
| 900 | | Have you ever heard/seen any message about adolescent nutrition on any of the following: | | |  | | |  |
| 901 | | Newspaper/magazine | Yes -----------------01  No ------------------02 | |  | | |  |
| 902 | | Radio | Yes -----------------01  No ------------------02 | |  | | |  |
| 903 | | TV | Yes -----------------01  No ------------------02 | |  | | |  |
| 904 | | Poster/ banner/ board | Yes -----------------01  No ------------------02 | |  | | |  |
| 905 | | Local theatre | Yes -----------------01  No ------------------02 | |  | | |  |
| 906 | | Local loudspeaker | Yes -----------------01  No ------------------02 | |  | | |  |
| 907 | | During a coffee ceremony | Yes -----------------01  No ------------------02 | |  | | |  |
| 908 | | Community/ village gathering (Edir, Equb ) | Yes -----------------01  No ------------------02 | |  | | |  |
| 909 | | Mobile phone (SMS) | Yes -----------------01  No ------------------02 | |  | | |  |
| 910 | | If yes, What do you remember from these messages?(do not read out list. select all mentioned.) | Eating a diversified diet---------------01  Taking iron-folic acid supplement---02  Taking deworming tablet --------------03 Other (specify) ------------------------99  ____________________ | |  | | | |

**Part 10. Adolescents’ dietary questions**

“I would like you to tell me what you had to eat or drink after you woke up yesterday morning. Did you eat that food at home?  What did you have next and at what time?” [Proceed through the day, repeating these questions as necessary, and record each food or drink consumed in column 3 of the 24-hour recall form. Remember to probe for any snacks and drinks consumed between meals]

**1001. Day of week (circle the day): 01-Mon 02-Tue 03-Wed 04-Thu 05-Fri 06-Sat 07-Sun**

| **Time** | **Place eaten** | **Types of**  **Food/drink** | **Description of ingredients** | **Amount consommed in local measurement** |
| --- | --- | --- | --- | --- |
|  |  |  |  |  |
|  |  |  |  |  |
|  |  |  |  |  |
|  |  |  |  |  |

102_02 Probe for sickness: 🞏 Yes 🞏 No

102_03 If yes, did sickness affect appetite? 🞏Yes 🞏 No
102_04 If yes, how? 🞏Increase 🞏decrease

102_05 Was food intake unusual? 🞏Yes 🞏No

102_06 If yes, how was it unusual?

102_07 Was it a feast day? 🞏Yes 🞏No

102_08 Was it a market day? 🞏Yes 🞏No

102_09 Was it a fasting day? 🞏Yes 🞏No

102_10 Probe for tablets: 🞏 Yes 🞏 No

802_11 🞏 Iron 🞏vitamins 🞏other supplements 🞏anti-malarial

**Part 11. Body Measurements (Height, weight and MUAC)**

|  | **Interviewer ID** | ________________ |
| --- | --- | --- |
| 1101 | Height in Centimeters (cm) | Reading 1 _____________  Reading 2 _____________  Average reading _______ |
| 1102 | Weight in Kilograms (kg) | Reading 1 _____________  Reading 2 _____________  Average reading _______ |
| 1103 | MUAC in Centimeters (cm) | Reading 1 _____________  Reading 2 _____________  Average reading _______ |

#

# አባሪዎች

# I. በጽሑፍ የቀረበ የስምምነት ቅጽ

| **መግቢያ** |
| --- |
| **መግቢያ/ዓላማ:**  ሄሎ፤ ውድ ተሳታፊ ስሜ: ________________ይባላል፡፡ እኔ የ3ኛ ዲግሪ ተማሪ የዮሴፍ ሀላላ የመመረቅያ ጥናት/ምርምር መረጃ ሰብሳቢ ነኝ፡፡  አቶ ዮሴፍ ሃላላ በደቡብ ኢትዮጵያ ክልል ውስጥ ባሉ ልጃገረዶች(10-19 ዕድሜ) ላይ የአመጋገብ ስርዓት፤ ደም ማነስ፣ የአመጋገብ አገልግሎት አጠቃቀም ችግርና ምቹ ሁኔታ ላይ ምርምር እያደረገ ነው፡፡  በጠቅላላ ዕድሜያቸው ከ10-19 ዓመት ክልል ውስጥ ያሉ **843** ልጃገረዶች በዚህ ጥናት ውስጥ ይሳተፋሉ፡፡ ተሳታፊ ለዚህ ጥናት የተመረጠው በአጋጣሚ ነው፡፡  **ሂደቶች፡** በዚህ ጥናት ለመሳተፍ ፍቃደኛ ከሆነች፤ ስለ ጤና ፣ ስለ አመጋገብ እና ስነ- ምግብ አገልግሎቶች እጠይቃለሁ:: ይህ ጥናት በአሥራዎቹ ዕድሜ ክልል ላይ የሚገኙ ልጃገረዶች እና ወላጆቻቸው ስነ-ህዝባዊ እና ኢኮኖሚያዊ ሁኔታ፤ የአመጋገብ ስርዓት፤ ባህሪ እና የህይወት ዘይቤዎች፤ የግል ንጽህና እና የአካባቢ ጤና አጠባበቅ ለማሻሻል ለምሰሩ አካላት ይጠቅማል፡፡  በጠቅላላ የመረጃ አሰባሰብ ሂደት 45 ደቂቃ ያህል ይፈጃል፡፡ በተጨማሪም የሰውነት ክብደት፤ ቁመት እና የላይኛው የእጅ ክንድ (MUAC) ልኬት እወስዳለሁ፡፡  ፍቃደኛ ከሆነች የላቦራቶር ቴክኒሽያን የደም ማነስ በሽታ ምርመራ ለማድረግ ከጣት ጫፍ የደም ናሙና ይወስዳል:: አነስተኛ የሄሞግሎቢን ደረጃ ያላቸው ልጃገረዶች (Hb <12 µg / dL) በአካባቢው ባለው ጤና ተቋማት ጋር ተገኝተው እንድታከሙ ያደረጋል፡፡  **አደጋ/ሥጋት:**  በዚህ ጥናት ውስጥ ከመሳተፉ የተነሳ ለጤንነት/ለቤተሰብ ምንም ስጋት እንደሌለ አምናለሁ፡፡ ምንም ስሜታዊ ጥያቄዎች የሉም ፡፡ ከጣት ጫፍ ደም ለመውሰድ በሹል ነገር ስወጋ ትንሽ ልያም ይችላል፤ ነገር ግን ወድሁኑ/በደቅቃዎች ውስጥ ይተዋል፡፡  **ጥቅሞች፡**  በዚህ ጥናት ውስጥ ከመሳተፍ የተነሳ የሚገኝ ቀጥተኛ ጥቅም ባይኖርም በአከባብ ለምገኙ የአመጋገብ ስርዓት እና የአካባቢ ጤና አጠባበቅ ለማሻሻል ለምሰሩ አካላት ይጠቅማል፡፡  **ካሳ:** በዚህ ምርምር ውስጥ ለተሳተፉ ተሳታፊዎች ምንም ክፍያ የለም፡፡ በዚህ ጥናት ውስጥ ስለተሳተፉ እናደንቃለን፡፡  **ምስጢራዊነት:** ምላሽ በተቻለ መጠን ምሥጢራዊ እናደርጋለን፡፡ የተሳታፊ ማንነት በሚስጢር ይጠበቃል፡፡ የጥናቱ ቡድን ኣባላት መረጃውን ብቻ ለማየት ይችላል፡፡ ስለ ተሳታፊ የተመዘገቡ መረጃዎችን የማግኘት እና አስፈላጊ ከሆነ እርማቶችን የማድረግ መብት አለ፡፡ ስምና የተሳታፊ ማንነት የምገልፁ ነገሮች በቅጾች ላይ አይጻፉም፡፡ ተመርማሪው መረጃዎችን ለምርምር ሥራ ብቻ ይጠቀማል፡፡  **የፈቃደኝነት ተሳትፎ እና መቋረጥ-** ተሳትፎ በፈቃደኝነት ይሆናል፡፡ ጥያቄዎቹን በከፊል ወይም ሙሉ በሙሉ ላለመመለስ ይቻላል፡፡ በማንኛውም ጊዜ ከጥናቱ የመውጣት መብት አለ፡፡ ትክክለኛ ወይም የተሳሳተ መልስ የለም፡፡ መሳተፍና ያለመሳተፍ ተሳታፊ ከጤና ተቁዋም ከምያገኝው ጥቅማጥቅም ጋር አይገነኝም፡፡  **የሚመለከተው አካል:**  ማነኛው ጥያቄ ካለ ለመመለስ ዝግጁ ነኝ፡፡ በተጨማር ዋና ተመራማሪ ከተፈለገ አቶ ዮሴፍ ሃላላ የጥናት ዋነኛ ተመራማሪ ስለሆኑ ማንኛውም ጥያቄ ቢኖር፤ በ +251913233179 መደወል ይቻላል፡፡  ስለ ጥናቱ ጥያቄ አለ? እኔ በምወስዳቸው ልከተቶች/መረጃዎች ላይ ጥያቄ ካለ የበለጠ ማብራሪያ ለመስጠት ዝግጁ ነኝ፡፡  **እድምያቸው 18-19 ዓመት ዕድሜ ክልል ላሉ ልጃገረዶች በጽሑፍ የቀረበ የስምምነት ቅጽ**  የጥናቱን ዓላማ እና አሠራር ተረድችያለሁ:: ለጥያቄዎች መልስ ለመስጠት እድል ተሰጥቶኛል፡፡ ተሳትፎ በፈቃደኝነት መሆኑን ተረድችያለሁ፡፡ ጥያቄዎቹን በከፊል ወይም ሙሉ በሙሉ ላለመመለስ እችላለሁ፡፡ በማንኛውም ጊዜ ከጥናቱ የመውጣት መብት አለኝ፡፡ ከጥናት ጋር ተያይዞ ጉዳት ሲደርስብኝ ስለሚከሰት ማካካሻ ወይም የህክምና አገለግሎት ተነግሮኛል::  በዚህ ጥናት ውስጥ ስለተሳተፍሽ እናደንቃለን፡፡ የአንቺ አስተያየት አስፈላጊ ስለሆነ ተሳታፊ እንደሚትሆኚ ተስፋ አደርጋለሁ፡፡  ለመሳተፍ ፈቃደኛ ከሆንሽ እባክሽ ከታች በተሰጠው ቦታ ላይ ፊርማሽን ወይም ጣትሽን አትሚ!  የተሳታፊው ፊርማ፡_______________ ቀን: ______________  ቃለ መጠይቅ አድራጊ ፊርማ: _______________ ቀን: __________ ለትብብርች እናመሰግናለን፡፡  **እድምያቸው 10-18 ዓመት ዕድሜ ክልል ውስጥ ያሉ ልጃገረዶች ላላቸው ወላጆች / አሳዳጊዎች በጽሑፍ የቀረበ የስምምነት ቅጽ**  የጥናቱን ዓላማ እና አሠራር ተረድችያለሁ:: ለጥያቄዎች መልስ ለመስጠት ለእኔና ለልጄ እድል ተሰጥቶኛል፡፡ ተሳትፎ በፈቃደኝነት መሆኑን ተረድችያለሁ፡፡ ጥያቄዎቹን በከፊል ወይም ሙሉ በሙሉ ላለመመለስ እንችላለን፡፡ በማንኛውም ጊዜ ከጥናቱ የመውጣት መብት አለን፡፡  በዚህ ጥናት ውስጥ ስለተሳተፉ እናደንቃለን፡፡ የእናንተ አስተያየት አስፈላጊ ስለሆነ ተሳታፊ እንደሚትሆኑ ተስፋ አደርጋለሁ፡፡  ለመሳተፍ ፈቃደኛ ከሆኑ እባክዎ ከታች በተሰጠው ቦታ ላይ ፊርማዎን ወይም ጣትዎን ያትሙ!  የተሳታፊው ፊርማ፡_______________ ቀን: ______________  ቃለ መጠይቅ አድራጊ ፊርማ: _______________ ቀን: _______________ ለትብብርዎ እናመሰግናለን፡፡  **እድምያቸው 10-18 ዓመት ዕድሜ ክልል ላሉ ልጃገረዶች በጽሑፍ የቀረበ የስምምነት ቅጽ**  ከወላጅ / ህጋዊ ሞግዚቶች ሙሉ ፈቃድን አግኝቻለሁ:: የአንቺ ፍቃደኝነትን እጠይቅሻለሁ፡፡  የጥናቱን ዓላማ እና አሠራር ተረድችያለሁ:: ለጥያቄዎች መልስ ለመስጠት እድል ተሰጥቶኛል፡፡ ተሳትፎ በፈቃደኝነት መሆኑን ተረድችያለሁ፡፡ ጥያቄዎቹን በከፊል ወይም ሙሉ በሙሉ ላለመመለስ እችላለሁ፡፡ በማንኛውም ጊዜ ከጥናቱ የመውጣት መብት አለኝ፡፡ ከጥናት ጋር ተያይዞ ጉዳት ሲደርስብኝ ስለሚከሰት ማካካሻ ወይም የህክምና አገለግሎት ተነግሮኛል::  የአንቺ አስተያየት አስፈላጊ ስለሆነ ተሳታፊ እንደሚትሆኚ ተስፋ አደርጋለሁ፡፡ በዚህ ጥናት ውስጥ ስለተሳተፍሽ እናደንቃለን፡፡  ለመሳተፍ ፈቃደኛ ከሆንሽ እባክሽ ከታች በተሰጠው ቦታ ላይ ፊርማሽን ወይም ጣትሽን አትሚ!  የተሳታፊው ፊርማ፡_______________ ቀን: ______________  ቃለ መጠይቅ አድራጊ ፊርማ: ______ ቀን: __________ ለትብብርች እናመሰግናለን፡፡ |

**አባሪ II. መጠይቅ**

| **ኮድ** | **ጥያቄ** | **ምላሽ** | | **ዝለል** |
| --- | --- | --- | --- | --- |
|  | **ክፍል 1. የዳሰሳ ጥናት መረጃ** |  | |  |
| 101 | የቃለ መጠይቅ አድራጊ ስም | _________________________ | |  |
| 102 | የተሳታፊ መለያ ቁጥር | ______________________ | |  |
| 103 | ቃለ መጠይቅ የተደረገበት ቀን | ------/------/--------- | |  |
| 104 | አድረሻ | ዞን______ወረዳ _______ቀበለ _____ጎጥ -------- | |  |
|  | **ክፍል 2: ስነ-ሕዝብ መረጃ** |  | |  |
| 201 | የልደት ቀንሽ መቸ ነው (ቀን/ወር/ ዓ.ም) | _______ | |  |
| 202 | እድሜሽ ስንት ነው? በዓመት | ______ | |  |
| 203 | ሐይማኖትሽ ምንድነው? | ኦርቶዶክስ----------01  ፕሮተስታንት ------02  ሙስልም---------03  ሌላ ካለ ግለጭ ..................................................99 | |  |
| 204 | በአሁኑ ጊዜ ትምህርት እየተማርሽ ነው? | አዎን--------01  አይደለሁም---------02 | |  |
| 205 | ያጠናቀቀሽው ከፍተኛ የትምህርት ደረጃ ምንድን ነው? | መደበኛ ት/ርት የለም-------------01  1-4 ክፍል---------02  5-8 ክፍል-----------03  9-10 ክፍል---------04  11-12 ክፍል------05  ኮሌጅ እና ዩኒቨርሲቲ--06 | |  |
| 206 | በአንድ ቤት ውስጥ ያለው የቤተሰብ መጠን ስንት ነው? | [__][__] (ቁጥር ጻፍ) | |  |
| 207 | የአባትሽ / የቤተሰብ ራስ ሥራ ምንድን ነው? | አርሶ አደር / የግብርና ሰራተኛ---01    የሰለጠነ ሰራተኛ -----02  ያልሰለጠነ ሰራተኛ---03  ንግድ / ነጋዴ ---------04  ተቀጣር ሰራተኛ-------05  ቤት ውስጥ ሰራተኛ ----06  ስራ አጥ--------07    ሌላ (ዝርዝር ግለጭ) .........................................99 | |  |
| 208 | የእናትሽ ስራ ምንድን ነው? | አርሶ አደር / የግብርና ሰራተኛ---01    የሰለጠነ ሰራተኛ -----02  ያልሰለጠነ ሰራተኛ---03  ንግድ / ነጋዴ ---------04  ተቀጣር ሰራተኛ-------05  ቤት ውስጥ ሰራተኛ ----06  ስራ አጥ--------07  ሌላ (ዝርዝር ግለጭ) ............................................99 | |  |
| 209 | የአባትሽ የትምህርት ደረጃ ምንድን ነው? | መደበኛ ት/ርት የለም-------------01  1-4 ክፍል---------02  5-8 ክፍል-----------03  9-10 ክፍል---------04  11-12 ክፍል------05  ኮሌጅ እና ዩኒቨርሲቲ--06 | |  |
| 210 | የእናትሽ የትምህርት ደረጃ ምንድን ነው? | መደበኛ ት/ርት የለም-------------01  1-4 ክፍል---------02  5-8 ክፍል-----------03  9-10 ክፍል---------04  11-12 ክፍል------05  ኮሌጅ እና ዩኒቨርሲቲ--06 | |  |
| 211 | የቤተሰብሽ አማካይ ወራዊ ገቢ ስንት ነው?  (ወላጆቻቸውን ጠይቅ) | ______________________ | |  |
|  | **ክፍል 3. የጤና ተቋም እና የአመጋገብ ስረዓት አገልግሎት ተዛማጅ ጥያቄዎች** | | |  |
| 301 | የጤና ኬላ ማእከል በቅርበት አላችው? | | አዎ ------01  የለም ------02 |  |
| 302 | የጤና ጣብያ ማእከል በቅርበት አላችው? | | አዎ ------01  የለም ------02 |  |
| 303 | ሆስፒታል በቅርበት አላችው? | | አዎ ------01  የለም ------02 |  |
| 304 | በቤታችውና በቅርበት ካለው ጤና ተቋማት መካከል ያለው ርቀት ስንት ኪ.ሜ. ነው? | | ------------------------ ኪ.ሜ  88. አላውቅም |  |
| 305 | በአቅራቢያው ወደሚገኘውን የጤና ተቋም ለመሄድ ምን ያህል ጊዜ ይፈጅብሻል? (አንድ ጉዞ) | | \|___\|____\|____\| በደቅቃ |  |
| 306 | በአቅራቢያው የሚገኘውን የጤና ተቋም ለመሄድ ምን መጓጓዣ ትጠቀማላችው? | | . በእግር ጉዞ----01  በተሽከርካሪ / በህዝብ መጓጓዣ---02  በቁሎ / ፈረስ----03  አላውቅም-----88 |  |
| 307 | በአቅራቢያው ያለውን የጤና ተቋማት ስላለው ጥቅም መረጃ አለሽ? | | አዎ ------01  የለም ------02 |  |
| 308 | አዎ ከሆነ ለጥያቄ 307፤ ዋናው የመረጃ ምንጭ ምንድነው? | | የጤና ባለሙያ-------------01  ጓደኛ፤ ጎረበት ------------02  ረድኦ፣ TV---------------03,  ሌላ ካለ ግለጪ------------------99 |  |
| 309 | በቤታችው ውስጥ በአመጋገብ አገልግሎት ዙርያ ውሳኔ ሰጪ ማን ነው? | | አባት ----01  እናት-----02  ሌላ ካለ ግለጪ-----------99 |  |
| 310 | የአመጋገብ ምክር / ትምህርት አገልግሎት በ6ወራት ውስጥ አግኝተሻል? | | አዎ ------01  የለም ------02 |  |
| 311 | ለ ጥያቄ 310 መልስ አዎን ከሆነ፤ የአመጋገብ ምክር / ትምህርት አገልግሎት የሰጡት ማን ናቸው? | | ጤና ኤክስቴንሽን ባለሙያ -----01  ነርስ/ሄልዝ አፍሰር --------02  ዶክተር -------03  ሌላ ካለ ግለጪ--------------------------99 |  |
| 312 | ለ ጥያቄ 310 መልስ አዎን ከሆነ፤ የአመጋገብ ምክር / ትምህርት አገልግሎት ያገኘሽው ከየት ነው? | | በጤና ተቁዋም(ጤና ጣቢያ/ ጤና ኬላ/ሆስፒታል)--01  ት/ቤት ---02  ቤት/ በማህበረሰብ ውስጥ---03  ሌላ ካለ ግለጪ------------------------------99 |  |
| 313 | በየ ስድስት ወሩ የፀረ-ተዋስያን መድኃንት ትወስጅያለሽ? (ክንኒ አሳይ) | | አዎ ------01  የለም ------02 |  |
| 314 | ለ ጥያቄ 313 መልስ አዎን ከሆነ፤ ስንት ክኒን በየ ስድስት ወሩ ትወስጅያለሽ? | | ------------------------------ቁጥሩን ግለጪ |  |
| 315 | ለ ጥያቄ 313 መልስ አዎን ከሆነ፤ የፀረ-ተዋስያን መድኃንት ከየት ነው ያገኘሽው? | | በጤና ተቁዋም(ጤና ጣቢያ/ ጤና ኬላ/ሆስፒታል)--01  ት/ቤት ---02  ቤት/ በማህበረሰብ ውስጥ---03  ሌላ ካለ ግለጪ-------------------------------------99 |  |
| 316 | አይሬን-ፎልክ አስድ ክንኒ እየወሰድሽ ነሽ ? (ክንኒ አሳይ) | | አዎ ------01  አይ ------02 |  |
| 317 | ለ ጥያቄ 312 መልስ አዎን ከሆነ፤ አይሬን-ፎልክ አስድ ክንኒ ከየት ነው ያገኘሽው? | | በጤና ተቁዋም(ጤና ጣቢያ/ ጤና ኬላ/ሆስፒታል)--01  ት/ቤት ---02  ቤት/ በማህበረሰብ ውስጥ---03  ሌላ ካለ ግለጪ-------------------------------------99 |  |
| 318 | የጤና እንክብካቤ አቅራቢዎች(ጤና ባለሙያዎች) ጥሩ የአመጋገብ ስርዓት አገልግሎት ይሰጣሉ ብለሽ ታስብያለሽ? | | አዎ ------01  አይ ------02 |  |
| 319 | ሳል /የመተንፈስ ችግር ባለፈው ሁለት ሳምንታት ዉስጥ አሞሽ ያዉቃል? | | አዎ ------01  አይ ------02 |  |
| 320 | ባለፈው ስድሰት ወራት ዉስጥ የደም ማነስ ምርመራ አስደርገሽ ታውቅያለሽ? | | አዎ ------01  አይ ------02 |  |
| 321 | ባለፈው ሁለት ሳምንታት ዉስጥ የተቅማጥ በሽታ አሞሽ ያዉቃል? | | አዎ ------01  አይ ------02 |  |
| 322 | በወባ በሽታ ታምመሽ ታውቂያለሽ? | | አዎ ------01  አይ ------02 |  |

## ክፍል 4: የልጃገረዶች ባህሪ እና የአኗኗር ዘይቤ

| 401 | ሲጋራ ታጨሽያለሽ? | | | አዎ ------01  አይ ------02 | መልስሽ ለጥያቄ 401 አይ ከሆነ ወደ ጥያቄ 403 እለፊ | |
| --- | --- | --- | --- | --- | --- | --- |
| 402 | አዎን ከሆነ፣ ለመጀመሪያ ጊዜ ሲጋራ ሲትሞክሪ ምን ያህል ዕድሜሽ ነበር? | | | ------------------ |  | |
| 403 | አልኮል መጠጥ ጠጥተሽ ታውቅያለሽ? | | | አዎ ------01  አይ ------02 | መልስሽ ጥያቄ ለ403 አይ ከሆነ ወደ ጥያቄ 405 እለፊ | |
| 404 | አዎን ከሆነ ለ403፤ አልኮል መጠጥ ቢያንስ ስንት ግዜ በ 7 ቀናት ውስጥ ጠጥተሻል? | | | አንድ ጊዜ-----01  ሁለት ጊዜ-----02  ሶስት ጊዜ እና ከዚያ በላይ -----03 |  | |
| 405 | ጫት ትቅምያለሽ ? | | | አዎ ------01  አይ ------02 | መልስሽ ለጥያቄ 405 አይ ከሆነ ወደ ጥያቄ 501 እለፊ | |
| 406 | አዎን ከሆነ፣ ለመጀመሪያ ጊዜ ጫት ሲትሞክሪ ምን ያህል ዕድሜሽ ነበር? | | | ----------------በአመት |  | |
| 407 | ጫት ቢያንስ ስንት ግዜ በ 7 ቀናት ውስጥ ትቅምያለሽ? | | | አንድ ጊዜ-----01  ሁለት ጊዜ-----02  ሶስት ጊዜ እና ከዚያ በላይ -----03 |  | |
| **ክፍል 5: የግል እና የአካባቢን ንፅህና መረጃ** | | | | | | |
| 501 | | መፀዳጃ ቤት አላችዉ? | አዎ ------01  የለም ------02 | | | መልስሽ ለጥያቄ 501 የለም ከሆነ ወደ ጥያቄ 503 እለፊ |
| 502 | | ለጥያቄ 501 አዎን ከሆነ፤ የትኛው የሽንት ቤት አይነት ነው? | **የውሃ መፀዳጃ ቤት**  ወደ ቧንቧ ማፍሰሻ መፀዳጃ ቤት---01  ወደ ፍሳሽ ቆሻሻ ማጠራቀምያ መፀዳጃ ቤት--02  ወደ ጉድጓድ ውስጥ የሚፈስ መፀዳጃ ቤት---03  ወደ ሌላ ቦታ የሚፈስ መፀዳጃ ቤት----04  ወደ ማይታወቅ ቦታ የሚፈስ መፀዳጃ ቤት----05  **ጎድጓድ መፀዳጃ**  በአየር የተሻሻለ ጉድጓድ መፀዳጃ ቤት-----06  ስላብ የተሰራ ጉድጓድ መፀዳጃ ቤት----07  ስላብ የልተሰራ ጉድጓድ መፀዳጃ ቤት----08  የኮምፎስት መፀዳጃ ቤት-----------09  የዱካ መፀዳጃ ቤት-----------10  ተንጠልጣይ መፀዳጃ ቤት----11  ቡሽ / መዳ ላይ መፀዳደት----12  ሌላ (ዝርዝር ግለጪ)---------------------------99 | | |  |
| 503 | | ለቤተሰብሽ አባላት ዋና የመጠጥ ውኃ ምንጭ ምንድነው? | **የቧንቧ ውሃ**  ወደ ቤት የገባ የቧንቧ ውሃ ------01  ወደ ግቢ የገባ የቧንቧ ውሃ --------02  ወደ ጎረቤት ግቢ የገባ የቧንቧ ውሃ ---03  ከግቢ ውጭ ያለው የቦኖ ውሃ-----04  ከጥልቅ ጉድጓድ የወጣ የቧንቧ ውሃ--05  **የጉድጓድ ውሃ**  የተጠበቀ የጉድጓድ ውሃ-----------06  ያልተጠበቀ የጉድጓድ ውሃ --------07  **የምንጭ ውሃ**  የተጠበቀ የምንጭ ውሃ------08  ያልተጠበቀ የምንጭ ውሃ----09  የዝናብ ውሃ -----------------10  በቦቲ የመጣ ውሃ -----------11  በትናንሽ ጋሪ የመጣ ውሃ ----12  **የገፀ-ምድረ ውሃ**  ወንዝ/ ዥረት---------------13  ኩሬ -----------------------14  የታሸገ ውሃ ---------------15  ሌላ ካለ ግለጪ_____________ 99 | | |  |
| 504 | | ውሃ ለማሰብሰብ/ለመቅዳት በእግር ጉዞ ምን ያህል ጊዜ ይወስዳል? (ደርሶ-መልስ) | ከ 5 የማይበልጥ/ ያነሰ--------01  5-15 ደቂቃ---------02  ከ15-30 ደቂቃዎች----------3  30 ደቂቃ - 1 ሰዓት-------04  ከ 1 ሰዓት በላይ---------05 | | |  |
| 505 | | ትላንት ሌሊት በቤታችሁ ስንት ሰዎች አድረዋል? | ____________________________ | | |  |
| 506 | | እንስሳት በቤት ውስጥ ይኖሩ ይሆን? | አዎ ------01  የለም ------02 | | |  |
| 507 | | የቤቶች ሁኔታ: የክፍሎች ቁጥር | ____________________________ | | |  |
| 508 | | የመስኮቶች ቁጥር | -------------------------- | | |  |
| 509 | | የወለል አይነት | ጭቃ /አፈር -------01  ስምንቶ -----02  ሌላ ካለ ግለጪ ___________________99 | | |  |
| 510 | | በየቀኑ ስንት ጊዜ ጥርስሽን ታጸጅያለሽ? | -------------------(ቁጥሩን አስገቢ) | | |  |
| 511 | | ከመብላትሽ በፊት እጅሽን ምን ያህል ግዜ ትታጠቢ ነበር? | በፍጹም-------01  አልፎ አልፎ------02  ዘወትር-----------03 | | |  |
| 512 | | ከመፀዳጃ ቤት በኋላ እጅሽን ትታጠብያለሽ? | አዎ ------01  አይ ------02 | | |  |
| 513 | | መልስሽ ለጥያቄ 512 አዎን ከሆነ፤ ከመፀዳጃ ቤት በኋላ ምን ያህል ግዜ እጅሽን ትታጠቢ ነበር? | በፍጹም-------01  አልፎ አልፎ------02  ዘወትር-----------03 | | |  |
| 514 | | እጅሽን ስትታጠብ ሳሙና ትጠቀምያለሽ? | አዎ ------01  አይ ------02 | | | መልስሽ ጥያቄ 512 አይ ከሆነ ወደ ጥያቄ 601 |
| 515 | | መልስሽ ለጥያቄ 514 አዎ ከሆነ.,፤ እጅሽን ስትታጠቢ ምን ያህል ጊዜ ሳሙና ትጠቀምያለሽ? | በፍጹም-------01  አልፎ አልፎ------02  ዘወትር-----------03 | | |  |

**ክፍል 6. ወጣት ሴት ልጆች የአመጋገብ ስርዓት መረጃ**

| 601 | ለቤተሰብ ዋና ምግብ ምንድ ነው? (ከአንድ በላይ መልስ ሊገኝ ይችላል) | ጤፍ ---01  በቆሎ-----02  ማሽላ-----03  ገብስ-----04  ስንዴ -----05  ሌላ የተለየ-----------99 |  |
| --- | --- | --- | --- |
| 602 | በአሁኑ ጊዜ እየተመገበ ያለው የምግብ ምንጭ ምንድነው? | ከቤት-------01  ከገበያ ግዢ----02  ከቤት እና ከገበያ ግዢ---03  ከዕርዳታ ------04  ከስጦታ -----05  ከብድር--------06  የተለየ ካለ-----------------99 |  |
| 603 | በቀን ስንት ግዜ ምግብ ትመገብያለሽ? | አንድ ጊዜ ---01  ሁለት ጊዜ-----02  ሶስት ጊዜ-------03  አራት ጊዜና ከዚያ በላይ----04 |  |
| 604 | መደበኛ ምግብሽን ዘልለሽ/ትተሸ ታውቅያለሽ? | አዎ ------01  አይ ------02 | መልስሽ ለጥያቄ 604 አይ ከሆነ ወደ ጥያቄ 701 |
| 605 | አዎ ከሆነ፤ የትኛው ምግብ ነው የሚዘለለው? | ቁርስ----01  ምሣ -----02  ዕራት----03  መክሰስ----04 |  |
| 606 | ቡና እና ሻይ ከምግብ ጋር ወንም በኋላ ትጠጭያለሽ? | ከምግብ በኋላ ------01  ከምግብ ጋር ------02 |  |
| 607 | መልስሽ ለጥያቄ 606 ከምግብ በኋላ ከሆነ፤ ከምግብ በኋላ ቡና እና ሻይ ስንት ደቂቃ ቆይተሽ ትጠጭያለሽ? | _______________________________________ |  |

**ክፍል 7-የምግብ ዋስትና እና አመጋገብ ጥያቄዎች**

**ለሚከተሉት ጥያቄዎች፤ ባለፉት 30 ቀናት ውስጥ ተሳታፊዋን ምን እንዳጋጠማት ጠይቂ**

| 701 | ባለፉት 30 ቀናት ውስጥ ቤተሰብሽ በቂ ምግብ ያለመኖሩ ተሰምቶሻል? | አዎን- 01  አይ -0 | መልስሽ "አይ" ከሆነ፣ ወደ >702 እለፊ |
| --- | --- | --- | --- |
| 701_1a | "አዎን" ከሆነ ለምን ያህል ጊዜ ነበር የተከሰተው? | አልፎ አልፎ (1-2 ጊዜ) - 01  አንዳንድ ጊዜ (ከ3-10 ጊዜ) - 02  ብዙ ጊዜ (ከ 10 ጊዜ በላይ) -03 |  |
| 702 | የሀብት/ገንዘብ እጥረት ምክንያት ባለፉት 30 ቀናት ውስጥ አንቺ ወይም ማንኛውም የቤተሰብ አባል የፈለጉትን ምግብ ሳይመገቡ ቀርተዋል? | አዎን- 01  አይ -0 | መልስሽ "አይ" ከሆነ, ወደ >703 እለፊ |
| 702_a | "አዎን" ከሆነ ለምን ያህል ጊዜ ነበር የተከሰተው? | አልፎ አልፎ (1-2 ጊዜ) - 01  አንዳንድ ጊዜ (ከ3-10 ጊዜ) - 02  ብዙ ጊዜ (ከ 10 ጊዜ በላይ) -03 |  |
| 703 | የሀብት/ገንዘብ እጥረት ምክንያት ባለፉት 30 ቀናት ውስጥ አንቺ ወይም ማንኛውም የቤተሰብ አባል ጥቅት የምግብ አይነት ተመግበዋል? | አዎን- 01  አይ -0 | መልስሽ "አይ" ከሆነ, ወደ >704 እለፊ |
| 703_a | "አዎን" ከሆነ ለምን ያህል ጊዜ ነበር የተከሰተው? | አልፎ አልፎ (1-2 ጊዜ) - 01  አንዳንድ ጊዜ (ከ3-10 ጊዜ) - 02  ብዙ ጊዜ (ከ 10 ጊዜ በላይ) -03 |  |
| 704 | አማራጭ ባለመኖሩ ምክንያት ባለፉት 30 ቀናት ውስጥ አንቺ ወይም ማንኛውም የቤተሰብ አባል መብላት የማትፈልጉትን ምግብ በልተዋል? | አዎን- 01  አይ -0 | መልስሽ "አይ" ከሆነ, ወደ >705 እለፊ |
| 704_a | "አዎን" ከሆነ ለምን ያህል ጊዜ ነበር የተከሰተው? | አልፎ አልፎ (1-2 ጊዜ) - 01  አንዳንድ ጊዜ (ከ3-10 ጊዜ) - 02  ብዙ ጊዜ (ከ 10 ጊዜ በላይ) -03 |  |
| 705 | ባለፉት 30 ቀናት ውስጥ አንቺ ወይም ማንኛውም የቤተሰብ አባል በቂ ምግብ ባለመኖሩ ምክንያት ትንሽ ምግብ መብላቱ ቅር ብሎሻል/ ተሰማሽ? | አዎን- 01  አይ -0 | መልስሽ "አይ" ከሆነ, ወደ >706 እለፊ |
| 705_a | "አዎን" ከሆነ ለምን ያህል ጊዜ ነበር የተከሰተው? | አልፎ አልፎ (1-2 ጊዜ) - 01  አንዳንድ ጊዜ (ከ3-10 ጊዜ) - 02  ብዙ ጊዜ (ከ 10 ጊዜ በላይ) -03 |  |
| 706 | በቂ ምግብ ባለመኖሩ ምክንያት ባለፉት 30 ቀናት ውስጥ አንቺ ወይም ማንኛውም የቤተሰብ አባል ጥቅት ምግብ (meals) በልቱዋል? | አዎን- 01  አይ -0 | መልስሽ "አይ" ከሆነ, ወደ >707 እለፊ |
| 706_a | "አዎን" ከሆነ ለምን ያህል ጊዜ ነበር የተከሰተው? | አልፎ አልፎ (1-2 ጊዜ) - 01  አንዳንድ ጊዜ (ከ3-10 ጊዜ) - 02  ብዙ ጊዜ (ከ 10 ጊዜ በላይ) -03 |  |
| 707 | የሀብት/ገንዘብ እጥረት ምክንያት ባለፉት 30 ቀናት ውስጥ በቤተሰብ ውስጥ ሙሉ በሙሉ ምግብ ጠፍቶ ያውቃል? | አዎን- 01  አይ -0 | መልስሽ "አይ" ከሆነ, ወደ >708 እለፊ |
| 707_a | "አዎን" ከሆነ ለምን ያህል ጊዜ ነበር የተከሰተው? | አልፎ አልፎ (1-2 ጊዜ) - 01  አንዳንድ ጊዜ (ከ3-10 ጊዜ) - 02  ብዙ ጊዜ (ከ 10 ጊዜ በላይ) -03 |  |
| 708 | በቂ ምግብ ባለመኖሩ ምክንያት ባለፉት 30 ቀናት ውስጥ አንቺ ወይም ማንኛውም የቤተሰብ አባል እየተራበ ባዶ አድሮ/ተኝቶ ያውቃል? | አዎን- 01  አይ -0 | መልስሽ "አይ" ከሆነ, ወደ >709 እለፊ |
| 708_a | "አዎን" ከሆነ ለምን ያህል ጊዜ ነበር የተከሰተው? | አልፎ አልፎ (1-2 ጊዜ) - 01  አንዳንድ ጊዜ (ከ3-10 ጊዜ) - 02  ብዙ ጊዜ (ከ 10 ጊዜ በላይ) -03 |  |
| 709 | በቂ ምግብ ባለመኖሩ ምክንያት ባለፉት 30 ቀናት ውስጥ አንቺ ወይም ማንኛውም የቤተሰብ አባል ሙሉ ቀን ምንም ምግብ ሳይበላ ቆይቶ ያውቃል? | አዎን- 01  አይ -0 |  |
| 709_a | "አዎን" ከሆነ ለምን ያህል ጊዜ ነበር የተከሰተው? | አልፎ አልፎ (1-2 ጊዜ) - 01  አንዳንድ ጊዜ (ከ3-10 ጊዜ) - 02  ብዙ ጊዜ (ከ 10 ጊዜ በላይ) -03 |  |

**ክፍል 8. የአመጋገብ ትምህርት ሁኔታ**

| **ተ.ቁ.** | **ጥያቄዎች** | | | **ምርጫ / መልስ** | | **ይዝለሉ** |  |
| --- | --- | --- | --- | --- | --- | --- | --- |
| 801 | ባለፉት ሦስት ወራት ውስጥ በቤታችው በጤና ኤክስቴንሽን ሰራተኛ ተጎብኝተሻል? | | | አዎን- 01  አይ -02  አላስታውስም -88 | | >806  >806 |  |
| 802 | ባለፉት ሦስት ወራት ውስጥ በቤታችው በጤና ኤክስቴንሽን ሰራተኛ ስንት ጊዜ ተጎብኝተሻል? | | | አንድ ጊዜ -01  ሁለት ጊዜ- 02  ሦስት ጊዜ -03  አራት ወይም ከዚያ በላይ ጊዜ- 04  አላስታውስም -88 | |  |  |
| 803 | የጤና ኤክስቴንሽን ሰራተኛ በቤታችው የመጨረሻ ጉብኝት ያደረገችበት ጊዜ መቼ ነበር? | | | ባለፈው 1 ወር ውስጥ -01  ከ 1-3 ወራት በፊት -02  ከ3-6 ወራት በፊት -03  አላስታውስም / አላውቅም -88 | |  |  |
| 804 | የጤና ኤክስቴንሽን ሰራተኛ በቤታችው የመጨረሻ ጉብኝት ስታደርግ የልጃገረዶች አመጋገብ ሁኔታ ተናግራ ነበር? | | | አዎን- 01  አይ -02 | | >806 |  |
| 805 | የጤና ኤክስቴንሽን ሰራተኛ በቤታችው የመጨረሻ ጉብኝት ስታደርግ የልጃገረዶች አመጋገብ በተመለከተ የተናገረችው ምን ነበር?(ከአንድ በላይ ምላሾች ተፈቅደዋል) | | | የተለያዩ የምግብ አይነቶችን መመገብ -01  የደም ማነስ መድኃኒት መውሰድ -02  የሆድ ውስጥ ትላትል መድኃኒት መውሰድ -03  ሌላ ካለ ግለጪ_____________ -04  __________________________ | |  |  |
| 806 | በማህበረሰቡ ውስጥ የጤና ኤክስቴንሽን ሰራተኛ (ባለፉት ሶስት ወራት ውስጥ) ተገነኝተሽ ታውቂያለሽ? | | | አዎን- 01  አይ -02  አላስታውስም -88 | | >811  >811 |  |
| 807 | ባለፉት ሦስት ወራት ውስጥ በማህበረሰቡ ውስጥ በጤና ኤክስቴንሽን ሰራተኛ ስንት ጊዜ ተጎብኝተሻል? | | | አንድ ጊዜ -01  ሁለት ጊዜ- 02  ሦስት ጊዜ -03  አራት ወይም ከዚያ በላይ ጊዜ- 04  አላስታውስም -88 | |  |  |
| 808 | በማህበረሰቡ ውስጥ የጤና ኤክስቴንሽን ሰራተኛ (ባለፉት ሶስት ወራት ውስጥ) የተገነኘሽው የት ነበር? | | | በመንደር ስብሰባ ላይ -01  በ ሴፍትነት ክፍያ ቀን-02  በሃይማኖት ቦታ/ተቁዋም -03  በአገልግሎት አቅራቢዎች(outreach) ወቅት -04  ሌላ ካለ ግለጪ_____________ 05  _____________________________ | |  |  |
| 809 | የጤና ኤክስቴንሽን ሰራተኛ በማህበረሰብ ውስጥ የመጨረሻ ጉብኝት ስታደርግ የልጃገረዶች አመጋገብ ሁኔታ ተናግራ ነበር? | | | አዎን- 01  አይ -02 | | >1211 |  |
| 810 | የጤና ኤክስቴንሽን ሰራተኛ በማህበረሰብ ውስጥ የመጨረሻ ጉብኝት ስታደርግ የልጃገረዶች አመጋገብ በተመለከተ የተናገረችው ምን ነበር?(ከአንድ በላይ ምላሽ ተፈቅደዋል) | | | የተለያዩ የምግብ አይነቶችን መመገብ -01  የደም ማነስ መድኃኒት መውሰድ -02  የሆድ ውስጥ ትላትል መድኃኒት መውሰድ -03  ሌላ ካለ ግለጪ -04  _______________________________ | |  |  |
| 811 | ባለፉት ሦስት ወራት ውስጥ በቤታችው በጤና ልማት ሠራዊት መሪ ተጎብኝተሻል? | | | አዎን- 01  አይ -02  አላስታውስም -88 | | >1216  >1216 |  |
| 812 | ባለፉት ሦስት ወራት ውስጥ በቤታችው የጤና ልማት ሠራዊት መሪ ስንት ጊዜ ተጎብኝተሻል? | | | አንድ ጊዜ -01  ሁለት ጊዜ- 02  ሦስት ጊዜ -03  አራት ወይም ከዚያ በላይ ጊዜ- 04  አላስታውስም -88 | |  |  |
| 813 | የጤና ልማት ሠራዊት መሪ በቤታችው የመጨረሻ ጉብኝት ያደረገችበት ጊዜ መቼ ነበር? | | | ባለፈው 1 ወር ውስጥ -01  ከ 1-3 ወራት በፊት -02  ከ3-6 ወራት በፊት -03  አላስታውስም / አላውቅም -88 | |  |  |
| 814 | የጤና ልማት ሠራዊት መሪ በቤታችው የመጨረሻ ጉብኝት ስታደርግ የልጃገረዶች አመጋገብ ሁኔታ ተናግራ ነበር? | | | አዎን- 01  አይ -02 | | >816 |  |
| 815 | የጤና ልማት ሠራዊት መሪ በቤታችው የመጨረሻ ጉብኝት ስታደርግ የልጃገረዶች አመጋገብ በተመለከተ የተናገረችው ምን ነበር? (ከአንድ በላይ ምላሽ ተፈቅደዋል) | | | የተለያዩ የምግብ አይነቶችን መመገብ -01  የደም ማነስ መድኃኒት መውሰድ -02  የሆድ ውስጥ ትላትል መድኃኒት መውሰድ -03  ሌላ ካለ ግለጪ -04  _______________________________ | |  |  |
| 816 | ባለፉት ስድስት ወራት በማህበረሰቡ ውስጥ የተደረገውን የልጃገረዶች አመጋገብ ሁኔታ ተከታትለሽ ነበር? | | | አዎን- 01  አይ -02 | |  |  |
| 817 | ባለፉት ሦስት ወራት ውስጥ በት/ቤታችው የአመጋገብ ትምህርት ነበር? | | | አዎን- 01  አይ -02  አላስታውስም -88 | | >900  >900 |  |
| 818 | ባለፉት ሦስት ወራት ውስጥ የአመጋገብ ትምህርት ለምንያህል/ስንት ጊዜ ነበር? | | | አንድ ጊዜ -01  ሁለት ጊዜ- 02  ሦስት ጊዜ -03  አራት ወይም ከዚያ በላይ ጊዜ- 04  አላስታውስም -88 | |  |  |
| 819 | የአመጋገብ ትምህርት ስለ ምን ነበር? | | | የተለያዩ የምግብ አይነቶችን መመገብ -01  የደም ማነስ መድኃኒት መውሰድ -02  የሆድ ውስጥ ትላትል መድኃኒት መውሰድ -03  ሌላ ካለ ግለጪ-04  _______________________________ | |  |  |
| ክፍል 9. የመገናኛ ብዙሃን ሽፋን | | | | | | | |
| **ተ.ቁ.** | | **ጥያቄዎች** | **ምርጫ / መልስ** | | **ይዝለሉ** | | |
| 900 | | የልጃገረዶች አመጋገብ ሥርዓት በተመለከተ በሚከተሉት መገናኛ ብዙሃን ላይ አይተሸ/ሰምተሽ ከሆነ፡ | | |  | | |
| 901 | | በጋዘጣ | አዎን-----01  አይ ------02 | |  | | |
| 902 | | በራድኦ | አዎን-----01  አይ ------02 | |  | | |
| 903 | | መልሱ አዎን ከሆነ፣ ከራድኦ ምን ታስታውሽያለሽ | የተለያዩ የምግብ አይነቶችን መመገብ -01  የደም ማነስ መድኃኒት መውሰድ -02  የሆድ ውስጥ ትላትል መድኃኒት መውሰድ -03  ሌላ ካለ ግለጪ-04 | |  | | |
| 903 | | ቲቭ | አዎን----- 01  አይ -----02 | |  | | |
| 904 | | ፖስተር/ባነር/ሰለዳ | አዎን----- 01  አይ -----02 | |  | | |
| 905 | | በአከባብ ቤተ ተውኔት | አዎን----- 01  አይ -----02 | |  | | |
| 906 | | በአከባብ ድምጽ ማጉያ | አዎን----- 01  አይ -----02 | |  | | |
| 907 | | በአከባብ ቡና እየጠጣን | አዎን----- 01  አይ -----02 | |  | | |
| 908 | | በአከባብ እድር/እቁብ | አዎን----- 01  አይ -----02 | |  | | |
| 909 | | በሞባይል መልዕክት | አዎን----- 01  አይ -----02 | |  | | |

**ክፍል 10. የልጃገረዶች አመጋገብ ጥያቄዎች**

ትናንት ጠዋት ከእንቅልፍ በኋላ ምን እንደበላሽ ወይም ምን እንደጠጣሽ እንድነግርኝ እፈልጋለሁ፡፡ ያንን ምግብ በቤት ውስጥ ነው የበላሽው? በቀጣይ እና በየትኛው ሰዓት ነበር የበላሽው? እነዚህን ጥያቄዎች እንደአስፈላጊነቱ እየደጋገመሽ በ 24 ሰዓታት ውስጥ የተበሉ ምግቦችንና መጠጦችን አይነቱንና መጠኑን ጭምር መዝግቢ፡፡

1001. የሳምንት ቀን (ቀኑን ክበቢ): 01-ሰኞ 02- ማክሰኞ 03-ዕሮብ 04-ሐሙስ 05-አርብ 06-ቅዳሜ 07-እሁድ

| ሠዓት | ምግብ የተበላበት ቦታ | ምግብ/መጠጥ | የምግብ ዝርዝሮች እና የምግብ አቀራረብ (ምግብ የተሰራበት ዝረዝር ነገሮች) |
| --- | --- | --- | --- |
|  |  |  |  |
|  |  |  |  |
|  |  |  |  |
|  |  |  |  |
|  |  |  |  |
|  |  |  |  |
|  |  |  |  |
|  |  |  |  |

102_02 ታማ እንደሆነች አውጣጣ: አዎን- 01 አይ -02

102_03 አዎን ከሆነ፤ በሽታው በምግብ ፍላጎት ተፅእኖ አድረጉዋል? አዎን- 01 አይ -02
102_04 አዎን ከሆነ፤ እንዴት? 1. ቀንሱዋል 2. ጨምሩዋል

102_05 የምግብ አቅርቦት ያልተለመደ ነበር? አዎን- 01 አይ -02

102_06 አዎን ከሆነ፤ እንዴት ያልተለመደ ነበር?

102_07 የበዓል ቀን ነበር? አዎን- 01 አይ -02

102_08 የገበያ ቀን ነበር? አዎን- 01 አይ -02

102_09 የጾም ቀን ነበር? አዎን- 01 አይ -02

102_10 መድኃኒት ወስዳ እንደሆነች አውጣጣ: አዎን- 01 አይ -02

802_11 1. አይረን 2. ቫይታሚኖች 3. ሌሎች ተጨማሪ መድሃኒቶች 4. ፀረ-ወባ መድሃኒት

**ክፍል 11. ሰውነት ልኬቶች (ቁመት፤ ክብደት እና MUAC)**

|  | የቃለ መጠይቅ መለያ ቁጥር | └─┴─┴─┘ |
| --- | --- | --- |
| 1101 | ቁሜት በሴንቲሜትር (ሴ.ሜ) | ንባብ 1 ______ንባብ 2 _____ አማካይ ንባብ _____ |
| 1102 | ክብደት በኪሎግራም (ኪ.ግ.) | ንባብ 1 _____________ንባብ 2 ______አማካይ ንባብ _____ |
| 1103 | **MUAC** በሴንቲሜትር (ሴ.ሜ) | ንባብ 1 _____________  ንባብ 2 _____________አማካይ ንባብ ________ |
|  | **ክፍል 12. ባዮሎጂካል ናሙና** |  |
| 121 | ከጣት ጫፍ የደም ናሙና ተወሰደ? | አዎን----- 01  አይ -----02 |
| 122 | ለጥያቄ 121 አዎን ከሆነ፤ የልከት መሣሪያ ንባብ | ንባብ ________  በጣም አደገኛ <7 ሚሊ ግራም / ሊ-----------------  መካከለኛ 7-11 ሚሊ ግራም / ሊ ------------- |
