# Supplementary material for: Undernutrition and its determinants among adolescent girls in low land area of Southern Ethiopia
Source: PLoS One. 2021 Jan 12;16(1):e0240677. doi: 10.1371/journal.pone.0240677 (PMC7802945; doi:10.1371/journal.pone.0240677)
Supplement: Attachment — Submitted filename: PONE-D-19-32334_reviewer.pdf [file pone.0240677.s004.pdf]

PLOS ONE

Under nutrition and its determinants among adolescent girls in low land area of southern Ethiopia

--Manuscript Draft--

**Manuscript Number:**	PONE-D-19-32334
**Article Type:**	Research Article
**Full Title:**	Under nutrition and its determinants among adolescent girls in low land area of southern Ethiopia
**Short Title:**	Under nutrition and its determinants among adolescent girls in low land area of southern Ethiopia
**Corresponding Author:**	Yoseph Halala Handiso, Msc<br>Wolaita Sodo University<br>Sodo, ETHIOPIA
**Keywords:**	Adolescent girls, Under-nutrition, determinants, southern Ethiopia
**Abstract:**	Abstract<br>Background : Undernutrition is one of the most common causes of morbidity and mortality among adolescent girls worldwide, especially in South-East Asia and Africa. Even though adolescence is a window of opportunity to break the intergenerational cycle of undernutrition, adolescent girls are a neglected group. The objective of this study was to assess the nutritional status and associated factors among adolescent girls in the Wolaita and Hadiya zones of Southern Ethiopia.<br>Methods : A community-based cross-sectional study was conducted, and a multistage sampling method was used to select a sample of 843 adolescent girls. Anthropometric measurements were collected from all participants and entered in the WHO Anthro plus software for Z-score analysis. The data was analyzed using EPI-data 4.4.2 and SPSS version 21.0. The odds ratios for logistic regression along with a 95% confidence interval (CI) were estimated. A P- value < 0.05 was declared as the level of statistical significance.<br>Result : Thinness and stunting are found to be public health problems in the study area. Age [AOR(adjusted odds ratio) (95% CI) = 2.91 (2.03-4.173)], family size [AOR (95% CI) = 1.63(1.105-2.396)], monthly income [AOR (95% CI) = 2.54(1.66-3.87)], taking deworming tablets [AOR (95% CI) = 1.56(1.11--21)], the father's educational status [AOR (95% CI) = 2.45(1.02-5.86)], skipping regular meals [AOR (95% CI) = 2.83(1.92-4.17)], the source of food for the family [AOR (95% CI) = 5.14(2.1--12.8)], visits from health extension workers [AOR (95% CI) = 1.72(1.7-2.4)], and hand washing before eating and after using the toilet [AOR (95% CI) = 2.25(1.079-4.675)] were associated with nutritional status in the Wolaita and Hadiya zones, Southern Ethiopia. An inter-sectorial collaboration should be used to implement community based health and nutrition programs, i.e. schools and agricultural experts should work together with health experts to improve the nutritional status of adolescent girls.
**Order of Authors:**	Yoseph Halala Handiso, Msc
	Tefera Belachew
	Cherinet Abuye
	Abdulhalik Workicho
	Kaleab Baye
**Additional Information:**	
**Question**	**Response**
**Financial Disclosure**<br><br>Enter a financial disclosure statement that describes the sources of funding for the	Addis Ababa center of Food Science and Nutrition, Wolaita Sodo university and Tufts University Ethiopia, for their small research grant

Powered by Editorial Manager® and ProduXion Manager® from Aries Systems Corporation

work included in this submission. Review the submission guidelines for detailed requirements. View published research articles from *PLOS ONE* for specific examples.<br><br>This statement is required for submission and **will appear in the published article** if the submission is accepted. Please make sure it is accurate.<br><br>**Unfunded studies**<br>Enter: *The author(s) received no specific funding for this work.*<br><br>**Funded studies**<br>Enter a statement with the following details:<br>• Initials of the authors who received each award<br>• Grant numbers awarded to each author<br>• The full name of each funder<br>• URL of each funder website<br>• Did the sponsors or funders play any role in the study design, data collection and analysis, decision to publish, or preparation of the manuscript?<br>• **NO** - Include this sentence at the end of your statement: *The funders had no role in study design, data collection and analysis, decision to publish, or preparation of the manuscript.*<br>• **YES** - Specify the role(s) played.<br><br>* typeset	
**Competing Interests**<br><br>Use the instructions below to enter a competing interest statement for this submission. On behalf of all authors, disclose any competing interests that could be perceived to bias this work—acknowledging all financial support and any other relevant financial or non-financial competing interests.<br><br>This statement **will appear in the published article** if the submission is accepted. Please make sure it is accurate. View published research articles from *PLOS ONE* for specific examples.	All authors have declared that no competing interests exist

**NO authors have competing interests**<br><br>Enter: *The authors have declared that no competing interests exist.*<br><br>**Authors with competing interests**<br><br>Enter competing interest details beginning with this statement:<br><br>*I have read the journal's policy and the authors of this manuscript have the following competing interests: [insert competing interests here]*<br><br>* typeset	
**Ethics Statement**<br><br>Enter an ethics statement for this submission. This statement is required if the study involved:<br><br>• Human participants<br>• Human specimens or tissue<br>• Vertebrate animals or cephalopods<br>• Vertebrate embryos or tissues<br>• Field research<br><br>Write "N/A" if the submission does not require an ethics statement.<br><br>General guidance is provided below. Consult the submission guidelines for detailed instructions. **Make sure that all information entered here is included in the Methods section of the manuscript.**	N/A

**Format for specific study types**<br><br>**Human Subject Research (involving human participants and/or tissue)**<br>• Give the name of the institutional review board or ethics committee that approved the study<br>• Include the approval number and/or a statement indicating approval of this research<br>• Indicate the form of consent obtained (written/oral) or the reason that consent was not obtained (e.g. the data were analyzed anonymously)<br><br>**Animal Research (involving vertebrate animals, embryos or tissues)**<br>• Provide the name of the Institutional Animal Care and Use Committee (IACUC) or other relevant ethics board that reviewed the study protocol, and indicate whether they approved this research or granted a formal waiver of ethical approval<br>• Include an approval number if one was obtained<br>• If the study involved *non-human primates*, add *additional details* about animal welfare and steps taken to ameliorate suffering<br>• If anesthesia, euthanasia, or any kind of animal sacrifice is part of the study, include briefly which substances and/or methods were applied<br><br>**Field Research**<br><br>Include the following details if this study involves the collection of plant, animal, or other materials from a natural setting:<br>• Field permit number<br>• Name of the institution or relevant body that granted permission	
**Data Availability**<br><br>Authors are required to make all data underlying the findings described fully available, without restriction, and from the time of publication. PLOS allows rare exceptions to address legal and ethical concerns. See the PLOS Data Policy and FAQ for detailed information.	Yes - all data are fully available without restriction

Powered by Editorial Manager® and ProduXion Manager® from Aries Systems Corporation

A Data Availability Statement describing where the data can be found is required at submission. Your answers to this question constitute the Data Availability Statement and **will be published in the article**, if accepted.<br><br>**Important:** Stating 'data available on request from the author' is not sufficient. If your data are only available upon request, select 'No' for the first question and explain your exceptional situation in the text box.<br><br>Do the authors confirm that all data underlying the findings described in their manuscript are fully available without restriction?	
**Describe where the data may be found in full sentences. If you are copying our sample text, replace any instances of XXX with the appropriate details.**<br><br>• If the data are **held or will be held in a public repository**, include URLs, accession numbers or DOIs. If this information will only be available after acceptance, indicate this by ticking the box below. For example: *All XXX files are available from the XXX database (accession number(s) XXX, XXX.*).<br>• If the data are all contained **within the manuscript and/or Supporting Information files**, enter the following: *All relevant data are within the manuscript and its Supporting Information files.*<br>• If neither of these applies but you are able to provide **details of access elsewhere**, with or without limitations, please do so. For example:<br><br>*Data cannot be shared publicly because of [XXX]. Data are available from the XXX Institutional Data Access / Ethics Committee (contact via XXX) for researchers who meet the criteria for access to confidential data.*<br><br>*The data underlying the results presented in the study are available from (include the name of the third party*	All relevant data are within the manuscript and its supporting information files

and contact information or URL).<br>• This text is appropriate if the data are owned by a third party and authors do not have permission to share the data.<br><br>* typeset	
Additional data availability information:	

Manuscript-edited and cleaned Click here to access/download;Manuscript;Manuscript2-edited and cleaned.docx

Undernutrition and Its Determinants among Adolescent Girls in Low Land Areas of Southern Ethiopia

Yoseph Halala[1], Tefera Belachew[3], Cherinet Abuye[4], Abdulhalik Workicho[3], Kaleab Baye[2]

1. School of Public Health, College of Health Sciences and Medicine, Wolaita Sodo University, Ethiopia yosefhalala@gmail.com
2. Center for Food Science and Nutrition, College of Natural and Computational Sciences Addis Ababa University, Honorary Research Fellow, Bioversity International and Deputy Regional Secretary for Africa, World Public Health Nutrition Association (WPHNA), kaleabbaye@gmail.com
3. Human nutrition unit, College of public health & Medical Science, Jimma University, Ethiopia, teferabelachew@gmail.com
4. ENGINE/Save the Children, Addis Ababa, Ethiopia, cherinet.abuye@savethechildren.org.

Corresponding Author: Yoseph Halala

Email: yosefhalala@gmail.com

All authors have contributed to this work.

Abstract

Background: *Undernutrition is one of the most common causes of morbidity and mortality among adolescent girls worldwide, especially in South-East Asia and Africa. Even though adolescence is a window of opportunity to break the intergenerational cycle of undernutrition, adolescent girls are a neglected group. The objective of this study was to assess the nutritional status and associated factors among adolescent girls in the Wolaita and Hadiya zones* of *Southern Ethiopia.*

Methods: *A community-based cross-sectional study was conducted, and a multistage sampling method was used to select a sample of* ***843*** *adolescent girls. Anthropometric measurements were collected from all participants and entered in the WHO Anthro plus software for Z-score analysis. The data was analyzed using EPI-data 4.4.2 and SPSS version 21.0. The odds ratios for logistic regression along with a 95% confidence interval (CI) were estimated. A P- value < 0.05 was declared as the level of statistical significance.*

Result: *Thinness and stunting are found to be public health problems in the study area. Age [AOR(adjusted odds ratio) (95% CI) = 2.91 (2.03-4.173)], family size [AOR (95% CI) = 1.63(1.105-2.396)], monthly income [AOR (95% CI) = 2.54(1.66-3.87)], taking deworming tablets [AOR (95% CI) = 1.56(1.11--21)], the father's educational status [AOR (95% CI) = 2.45(1.02-5.86)], skipping regular meals [AOR (95% CI) = 2.83(1.92-4.17)], the source of food for the family [AOR (95% CI) = 5.14(2.1--12.8)], visits from health extension workers [AOR (95% CI) = 1.72(1.7-2.4)], and hand washing before eating and after using the toilet [AOR (95% CI) = 2.25(1.079-4.675)] were associated with nutritional status in the Wolaita and Hadiya zones, Southern Ethiopia. An inter-sectorial collaboration should be used to implement community based health and nutrition programs, i.e. schools and agricultural experts should work together with health experts to improve the nutritional status of adolescent girls.*

Introduction

Adolescence is defined as the age range from 10–19 years, and it is a period of transition from childhood to adulthood. The adolescent age group comprises 20% of the global population [1]. Malnutrition, particularly undernutrition, is highly prevalent among adolescents in developing countries [2, 3]. A study from northern Ethiopia reported high levels of stunting (26.5%) and thinness (58.3%) among adolescents [4]. Nutrition status among adolescents is an important determinant of health outcomes; undernutrition affects the health status of adolescent girls. In addition to causing significant mortality, it creates long lasting effects on the growth, development, and physical fitness of survivors [5]. This, in turn, affects their ability to learn and work at maximal productivity [6]. Undernutrition is an indicator of poor nutrition and has major consequences on human health as well as the social and economic development of the population [7]. Physical growth and development during puberty increase requirements for energy, protein, and many vitamins and minerals, and deficiencies can lead to physiological, anatomical, and functional disturbances [8]

The nutritional status of adolescent girls can have intergenerational effects because adolescent girls with poor nutritional status are more likely to give birth to low birth weight infants [8, 9]. Focus on adolescent girls is important because their health and nutritional status before as well as during pregnancy influences fetal growth and newborn health. Adolescent girls' health and undernutrition is an important determinant of adverse fetal outcomes, including low birth weight, preterm births, stillbirths, and an increased risk of neonatal mortality [10]. Therefore, adequate nutrition is key; it is associated with a better quality of life and has many intergeneration benefits[11].

Most causes of malnutrition are related to poor care, poor economic status, and food insecurity; however, malnutrition can sometimes be inherited genetically [12]. Family size, presence of malaria

infections, cigarette smoking, alcohol and drug use, environmental pollution, and domestic violence are predictors of undernutrition [13].

In regions of South-East Asia and Africa, a large number of adolescent girls suffer from chronic undernutrition, which adversely impacts their own health and development, as well as that of their offspring, contributing to an intergenerational cycle of malnutrition [14].

Even though the sustainable development goals (SDGs) include an adolescent nutrition service which is addressing adolescent malnutrition, the nutritional status of adolescent girls is not improving [15]. The government of Ethiopia officially launched the National Nutrition Program (NNP) in 2009, which aimed to reduce malnutrition in Ethiopia by integrating adolescents' nutrition into community-based health and development programs but faced many challenges. The Ethiopian NNP II (2016-2020) incorporated initiatives to improve the nutritional status of adolescent girls, but which interventions under which circumstances are effective remains unknown [16, 17]. However, most of these studies have included only adolescents attending school, and thus, the results of these studies cannot be generalized to the whole adolescent community. In addition to this, there are no community based studies conducted in Southern Ethiopia among adolescent girls. Therefore, understanding nutritional status and its associated factors are critical to timely address malnutrition in this age group.

Methods a[illegible] Materials

The study was conducted in the Wolaita and Hadiya zones of Southern Ethiopia. These zones are predominantly dependent on agriculture, practicing mixed crop-livestock production and living in permanent settlements. Within their landholdings, community members cultivate fruits, vegetables, roots, and tuber crops.

Fig 1 Map of the study sites (Wolaita and Hadiya zones) in southern nation nationality and peoples region (SNNPR)2019

A community-based cross-sectional study was conducted at two zones in Southern Ethiopia from April 30, 2019 to May 30, 2019. The inclusion criteria were adolescent girls (both attending and not attending school) between the ages of 10-19 years in two Southern Ethiopian zones. Participants who met the inclusion criteria were randomly selected to be the study population. Body mass index for age z-score and height for age z-score were the dependent variables. Age, educational status of the participant, family size, maternal and paternal educational, access to nutritional counseling services in health facilities, deworming tablets, iron-folic acid supplementation, household monthly income, source of food, and number of meals per day were the independent variables for our study.

Sample size determination

A single population proportion formula, [$n=z_{\frac{\propto}{2}}^{2}P\ (1\text{-}P)\ /d^{2}$] was used to estimate the sample size. From the literature review, the prevalence of thinness (24.4%) and stunting (29.4%) were used for sample size calculations. For this study, stunting (29.4%) was used to estimate the sample size as it gives a larger sample; considering a 95% confidence interval (CI) and d=0.05%, the initial sample size was 383. By adding 10% for non-respondents and a design effect of 2.4, the final sample size was **843**. $n= \underline{(Z_{\alpha/2})^{2} * p\ (1\text{-}p)\ DE\ /}d^{2}$. Where: Z = Standard normal distribution value at 95% CI = $(1.96)^2$, DE = design effect, and d = 0.05 (5% margin of error)

Sampling procedures

This study used multistage sampling techniques and was conducted in the Wolaita and Hadiya zones. From these two selected zones, two districts were selected based on a simple random sampling procedure, the Humbo district from Wolaita zone and the Misrak Badawacho district from the Hadiya zone. Three kebeles were selected from each district using a simple random method. A census of

adolescent girls was conducted at these selected kebeles. This census was developed with the help of both the local government administration, woreda in particular, and health extension workers. During the development of the census, if there was more than one adolescent girl in a household, one adolescent girl was randomly selected. From the selected six kebeles, 843 participants were chosen depending on the number of adolescent girls in each kebele. Participants were drawn from each kebele based on probability proportional to size (PPS) sampling. The sampling techniques depended on the number of adolescent girls in each kebele. Adolescent girls who were not eligible for the study were excluded.

Anthropometric measurements

Anthropometrics (i.e., height and weight) were measured on all sampled adolescent girls. Weight was measured to the nearest 100 g using a standard SECA digital scale while the participants wore light clothing and no shoes. The scale was calibrated after weighing each participant. Height was measured in a standing position to the nearest 0.1 cm using a vertical board with a detachable sliding headpiece. Body mass index for age z-scores and height for age z-scores were calculated using the height, weight, and age of the participants.

Data collection

A structured interviewer-administered questionnaire was used to collect data. The questionnaire was developed based on a thorough review of the current literature. A total of eight nurses with BSc degrees; previous experience collecting data; and knowledge of the culture, language, and norms of the community were employed to collect data using a pretested structured questionnaire. In addition to this, two supervisors with MSc's in public health were employed to supervise the data collection process. Data was collected on weekends for adolescent girls who attended school during the weekdays. The principal investigator controlled the daily overall study activities.

Statistical analysis

First, the data were checked for completeness and consistency for data entry and cleaning. Then, data were entered into the computer using EPI-data version 4.4.2 and exported to SPSS version 21.0 for further analysis. Descriptive statistics such as frequencies, proportions, and cross-tabulation were used to present the data. In addition, bivariate logistic regression analysis was performed to assess the association between independent and dependent variables. Variables that showed an association (p-value ≤ 0.25) in the bivariate analysis were included in the final multivariate logistic regression model. Odds ratios for logistic regression along with a 95% CI were estimated. A p-value less than 0.05 was declared statistically significant.

Data quality assurance

The questionnaire was prepared in English, translated to Amharic, and rendered back to English to maintain consistency of the questions. Data collectors and supervisors were trained for 4 days to properly fill out the questionnaire and measure anthropometry. Data collectors were selected from each zone so they could communicate fluently in the local language and understand the socio-cultural practices of the community. The questionnaire was pre-tested on 5% adolescent girls in a similar area to the study sites to ensure reliability. Feedbacks from the pre-test were incorporated into the final questionnaire design. Principal investigators and supervisors performed checks on the spot and reviewed all the completed questionnaires to ensure completeness and consistency of the information collected.

Standardization of anthropometric measurements was conducted. To standardize anthropometric measurements, during training an expert took two heights and weight measurements for ten adolescent girls and then let each data collector take the measurements for all ten girls twice. Then, the averages of the two measurements for each adolescent girl taken by the data collector were compared with the average of the expert's measurements. The technical error of measurement and coefficient of variance

(CV) were computed for all data collectors using E[illegible] for SMART software. Data collators with unacceptable TEM and CV c[illegible] repeat the steps again.

Ethical considerations

The study was approved by Addis Ababa University (AAU), College of Natural Sciences Research Ethics Review Committee. The official letter of cooperation was written to the Wolaita and Hadiya zones, and the district of health offices. The nature of the study was fully explained to the study participants and parents/guardians. Informed verbal and written consents were obtained from the parents/guardians for adolescent girls a[illegible] < 18 years old and assent was obtained from the participant before the interview. Participants ≥ 18 years were asked to provide verbal and written consent. The collected data were kept confidential. Each participant was given a code number, and the data were stored in a secure and password-protected database.

Results

Socio-demographic characteristics of adolescent girls in Southern Ethiopia

Eight hundred and twenty adolescent girls participated with a response rate of 97.3%.

As shown in Table 1, the average age of the study participants was 14.[illegible]1.9) years, the average family size was [illegible] (±1.83) persons, while 69.3% of the households had [illegible] family members and 30.7% had [illegible] family members. About three fourths (70%) of the study participants were in grades [illegible] 8, 23.3% were in grades 1-4 , and only 0.5% had no formal education. Most of the study participants were Protestant (7[illegible]%), but [illegible]% were Orthodox Christian, and only 0.7% were Muslims. Ab[illegible] 33.4% of the study participants were from households that have < 1000 ETBir[illegible]onthly income and 30.3% are from households that have > 2000 ET[illegible]r monthly income.

Table 1 [illegible]ocio-demographic characteristics of adolescent girls in Southern Ethiopia, 2019

Variable	Le[illegible]	Frequency(n)	Percent (%)
Age	10–13	393	47.9
	14–16	373	45.5
	17–19	54	6.6
Educational status	No formal education	4	0.5
	1–8 grade	765	93.3
	9–12 grade	49	6.0
	College and University	2	0.2
Religion	Orthodox	281	34.3
	Protestant	533	65.0
	Muslim	6	0.7
Family size	≤5 family members	252	30.7
	>5 family members	568	69.3
Monthly household income	<1000 ETBirr	274	33.4
	1001–2000 ETBirr	298	36.3
	>2000 ETBirr	248	30.3

Source: f[illegible] survey, 2019

Nutrition service and health-related factors of adolescent girls in Southern Ethiopia

As indicated in Table 2, approxima[illegible] 70.4 % of the study participants did not receive nutrition education. Only 29.6 % of the study participants had nutrition education. Similarly, 54.9% of the study

participants never received deworming tablets and only 45.1% have received a deworming tablet. Out of the participants who have taken deworming tablets, 65.6% have taken two tablets and 34.4% have taken one tablet every six months. When considering iron and folate supplementation, only 0.4% of the study participants have supplements. Of the total study participants with access to nutrition services, only 60.4% received friendly nutrition service, but 39.5% of the study participants did not receive friendly nutrition service and they were not satisfied by the services that were provided by health experts. In 66.1% of the households, the fathers were the primary decision-makers regarding nutrition service. About 27.8% of the study participants had a cough in the two weeks before data collection.

Table 2. Nutrition service and health-related factors of adolescent girls in Southern Ethiopia, 2019

Variable	Level	Frequency(n)	Percent (%)
Decision maker for nutrition service	Father	542	66.1
	Mother	78	9.5
	Jointly(Mother & Father)	200	24.4
Received nutrition education	Yes	243	29.6
	No	577	70.4
Received deworming tablets	Yes	450	54.9
	No	370	45.1
Number of deworming tablet received	One	155	34.4
	Two	295	65.6
Received iron folic acid supplementation (IFAS)	Yes	3	0.4
	No	817	99.6
Friendly nutrition service given	Yes	495	60.4
	No	324	39.5
Presence of cough	Yes	228	27.8

	No	592	72.2

Source: Field survey, 2019; IFAS, = Iron- folic acid supplementation

Health and sanitation-related factors of adolescent girls in Southern Ethiopia

Table 3 describes the health and sanitation related conditions of the study participants. Of the total 820 subjects, 47% of the adolescent girls are living on the floors of houses that are made from mud, and 58.5% of the study participants are living with domestic animals in the same house. Similarly, 48.7% of the study participants are brushing their teeth once per day, 53.3% are washing their hands sometimes before eating their food, 41.7% are usually washing their hands before eating, 3.4% are not washing their hands at all, 90.1% are washing their hands after using the toilet, and 6.8% are not washing their hands at all after using the toilet. When washing their hands, 90.1% of the study participants are using soap and 9.9% are not using soap. Out of the total participants who are using soap when washing their hands, only 42% are usually using soap and 58% are sometime using soap.

Table 3. Health and sanitation-related factors of adolescent girls in Southern Ethiopia, 2019

Variable	Level	Frequency(n)	Percent (%)
Type of floor participants are living on	Cement	385	47.0
	Muddy	435	53.0
Animals living in the same house?	Yes	480	58.5
	No	340	41.5
Number of windows	0	4	0.5
	1	41	5.0
	2	231	28.2
	3	297	36.2
	5	11	1.3
Frequency of teeth brushing (times per day)	0	29	3.5
	1	399	48.7

	2	266	32.4
	3	123	15.0
Do you wash your hands before eating?	Not at all	28	3.4
	sometimes	437	53.3
	Usually	342	41.7
Do wash your hands after using the toilet?	Yes	764	93.2
	No	56	6.8
Are you using soap when washing your hands?	Yes	739	90.1
	No	81	9.9
How often do you use soap when washing your hands?	Sometimes	429	58.0
	Usually	310	42.0

Source: Field survey, 2019

Meal patterns of adolescent girls in Southern Ethiopia

As indicated, about 39.5% of the study participants are eating ≥ four times per day. This indicates 60.5% of the study participants are skipping regular meals. When considering the meals that participants are skipping, 27.6%, 1.8%, and 42% are skipping breakfast, lunch, and snack, respectively. Similarly, 41.6% of the study participants are eating smaller meals that do not satisfy their needs. Maize is the primary staple food for 40.6% of the study participants, and 38.8% consume both teff and maize as a staple food. Participants purchase food from the market (40.4%) or grow their own food (50.[illegible]%) (Table 4).

Table 4. Meal Patterns of adolescent girls in Southern Ethiopia, 2019

Variables	Level	Frequency(n)	Percent (%)
Number of meals per day	Two times	6	0.7
	Three times	490	59.8

	Four times and above	324	39.5
Skip regular meals	Yes	496	60.5
	No	324	39.5
Meal skipped	Breakfast	137	27.6
	Lunch	15	1.8
	Snack	344	42.0
Staple food	Teff	169	20.6
	Maize	333	40.6
	Teff & Maize	318	38.8
Where do you get food	Produce your own	414	50.5
	Market purchase	331	40.4
	Produce your own and market purchase	69	8.4
Eat small meals	Yes	341	41.6
	No	479	58.4

Source: Field survey, 2019

Nutritional status of adolescent girls in Southern Ethiopia

As shown in Table 5, 69.5% of the study participants have a normal body mass index i.e. body mass index for age z-score is between -2 and [illegible] From the total study participants, 19.5% are moderately thin as defined by a body mass index for age z-score between -2 and -3, 8% are severely thin as defined by a body mass index for age z-score [illegible] -3. Only 3% of the study participants are overweight. When we considered the stunting status of the study participants, 91.2% are normal as defined as a height for age z-score > -2, 7.8% are moderately stunted, and 1% are severely stunted.

Fig 2 Comparison of BMI-for-age z-scores (BAZ) of the study population (N=820) with the 2007 WHO growth reference populations

Fig 3. Comparison of height-for-age z-scores (HAZ) of the study population (N=820) with the 2007 WHO growth reference populations, 2019

Fig 4. BMI for age z-scores (BAZ) among adolescent girls in Southern Ethiopia, 2019.

Fig 5. Height for age z-scores (HAZ) among adolescent girls in Southern Ethiopia, 2019

Table 5. Nutritional status of adolescent girls in Southern Ethiopia, 2019

Variables	Level	Frequency(N)	Percent (%)
Grouped BAZ	BAZ between -2 and +1	570	69.5
	BAZ between -2 and -3	160	19.5
	BAZ < -3	65	8.0
	BAZ between +2 and +1	25	3.0
Grouped HAZ	HAZ > -2	748	91.2
	HAZ between -2 and -3	64	7.8
	HAZ < -3	8	1.0

Source: field survey, 2019; BAZ, BMI for age z-score; HAZ, height for age z-score

Association between variables and nutritional status of adolescent girls in Southern Ethiopia

The present study shows an association between some variables with nutritional status, as defined by BMI for age z-score (BAZ), of the study participants. BAZ was statistically associated with age, family size, monthly household income, receiving deworming tablet(s), educational status of the participant's fathers, decision making power for nutrition service, skipping regular meals, source of food, and receiving home visits from health extension workers (Table 6).

Table 6. Factors associated with nutritional status (BAZ) of adolescent girls in Southern Ethiopia, 2019

Variables	Level	BAZ (< [illegible])	BAZ (> -2)	Crude OR (CI)	Adjusted OR (CI)
Age (years)	10-14	144	243	2.397 (1.748-3.286)***	2.910 (2.030-4.173)***
	> 15	83	344	1	1
Family size	≤ 5	57	195	1	1
	> 5	170	398	1.46 (1.034-2.064)*	1.627 (1.105-2.396)*
Monthly income (ETBirr)	< 1000	126	148	3.37 (2.28-4.98)***	2.54 (1.66-3.87)***
	1000-2000	49	249	0.779 (0.504-1.205)	0.74 (0.475-1.158)
	> 2000	52	196	1	1

		Nutritional status			
Variables	Levels	BAZ (< -2)	BAZ (> -2)	Crude OR (CI)	Adjusted OR (CI)
Receiving deworming tablets	Yes	101	349	1	1
	No	126	244	1.8 (1.3-2.4)***	1.56 (1.1--21)*
Father's educational status	No formal education	30	51	1.94 (1.1-3.4)*	2.3 (1.1-4.8)*
	1-8 grade	73	190	1.28 (0.82-1.96)	1.7 (0.96-2.87)
	9-12 grade	81	210	1.27 (0.83-1.95)	1.78 (0.86-3.01)
	College and University	43	142	1	1

Decision-maker for nutrition service	Father	168	374	2.05 (1.37-3.07)**	1.89 (1.214-2.94)**
	Mother	23	55	1.905 (1.37-3.07)*	2.022 (1.016-4.024)*
	Jointly	36	164	1	1
Skipping regular meals	Yes	178	318	3.14 (2.2-4.48) ***	2.83 (1.92-4.17)***
	No	49	275	1	1

		Nutritional status			
Variables	Level	BAZ (< -2)	BAZ (> -2)	Crude OR (CI)	Adjusted OR (CI)
Visited by health extension worker regularly	Yes	83	303	1	1
	No	144	290	1.813 (1.32-2.483)***	1.72 (1.7--2.4)**
Source of family food	Produce own	109	306	3.74 (1.57-8.89)**	3.288 (1.3--8.1)*
	Market purchase	112	224	5.25 (2.21-12.5)***	5.14 (2.1--12.8)***
	Produce own and market purchase	6	63	1	1

**p-value* < 0.05, ***p-value* < 0.0[illegible], ****p-value*<0.0001; BAZ, BMI for age z-score

There is also an association between some variables with nutritional status, as defined by height for age z-scores (HAZ), of the study participants. HAZ of the study participants was statistically associated with decision making power for nutrition service, hand washing practice before eating and after using the toilet, and visiting a community health extension worker (Table 7).

Table 7. Factors associated with nutritional status (HAZ) of adolescent girls in Southern Ethiopia, 2019

	**Nutritional status**				
Variables	Level	**HAZ** (< -2)	**HAZ** (> -2)	Crude OR (CI)	Adjusted OR (CI)
Decision-maker for nutrition service	Father	54	488	2.656 (1.241-5.685)**	2.529 (1.106-6.087)*
	Mother	10	68	3.529 (1.4-9.310)**	2.58 (0.89-7.45)
	Jointly	8	192	1	1
Hand washing before eating and after toilet	Yes	61	703	1	1
	No	11	45	2.82 (1.39-5.73)**	2.25 (1.079-4.675)*
Visited by community health extension worker	Yes	13	237	1	1
	No	59	509	2.13 (1.14- 3.93)*	2.036 (1.059-3.914)*

**p-value* < 0.05, ***p-value* < 0.001, ****p-value*<0.0001; HAZ, height for age z-score

Discussion

Health and nutritional status of adolescent girls in Southern Ethiopia

The BAZs revealed that 19.5% of the study participants were moderately thin and 8% were severely thin. The prevalence of thinness is higher in our study than in a study conducted in the Amhara Region which indicates that 13.6 % of adolescent girls are thin. Similarly, 7.8% and 1% of our study participants are moderately stunted and severely stunted, respectively. This is lower than the study conducted in the Amhara Region which indicated that 31.5 % of adolescent girls are stunted [18]. Our study is also different from a study conducted in Adwa, Northern Ethiopia, which indicated that the prevalence of thinness and stunting was 21.4% and 12.2%, respectively [19]. Another study conducted in Northern Ethiopia indicated that 21.6% of adolescent girls are thin and 21.2% are stunted [9]. Our results are also lower than a study conducted in Bangladesh which indicated that the prevalence of thinness and stunting was 49.74% and 15.1%, respectively [20]. The reasons for the observed undernutrition of the current study participants might be due to their low monthly household income because 33.[illegible]% of the study participants were from the families whose monthly incomes were below 1000 ETBirr. Similarly, 69.3% of the study participants are from families with ≥ 5 people. In our study, 14.3% of the study participants' fathers have no formal education and 9.9% of the study participants have only completed grades 1-4. Only 41.7% of our study participants usually wash their hands before eating food. Also, 42.0% of our study participants usually use soap when washing their hands. A similar study, conducted in the Somali Region of Ethiopia, indicated that hand washing with soap after using the toilet and before eating affects the nutritional status of adolescent girls [21]. This might lead to the low nutritional status of adolescent girls. Moreover, 60.5% of the study participants are skipping regular meals. This finding is greater than the study conducted among adolescent girls in Agarfa High School, Oromia Region which indicated the prevalence of skipping meals was 49.53% [22]. In addition to skipping their regular meals, 40.4% of the study participants are purchasing their food from the market. In order to buy food from the market, cash

income is needed. This might be affected by market fluctuation, household income, and takes more time which can lead to undernutrition [23].

The decision-making power of the family also might affect the nutritional status of adolescent girls. Decision-making for receiving nutrition services is under the control of 66.1% of the study participants' father. Similarly, 45.1% of the study participants did not receive a deworming tablet. Therefore, this might further aggravate the low nutritional status of the study subjects [24].

Factors associated with the nutritional status (BAZ) of adolescent girls in Southern Ethiopia

In this study, the BAZs of the study participants was statistically associated with the age of the adolescent girls ($p<0.001$). Adolescent girls between the ages of 10-14 years were 2.9 times more likely to be malnourished (thin) than adolescent girls ≥ 15 years old. This finding is in line with the study conducted in the Amhara Region [18].

Family size was statistically associated with the nutritional status of adolescent girls ($p<0.05$). Adolescent girls with a family size > 5 were 1.6 times more likely to be malnourished (thin) than those who came from a family with ≤ 5 people. This finding is supported by studies conducted in the city of Arar [25], Nigeria [26], and the Amhara Region [18]. Large families may share food among the family members [19].

Monthly household income is statistically associated with the nutritional status of adolescent girls ($p<0.001$). Adolescent girls from families whose monthly income was < 1000 ETBirr were 2.5 times more likely to be malnourished (thin) than those from families who have monthly incomes > 2000 ETBirr. This finding is in line with studies conducted in Bangladesh [27, 28] and Nigeria [29]. This might be because the household income affects the purchasing power of the household and families with lower incomes are more likely to be malnourished [30].

Taking deworming tablets was significantly associated with the nutritional status of the study participants ($p<0.05$). Study participants who did not take deworming table every six months were 1.56 times more likely to be malnourished (thin) than those who took a deworming tablet every six months. According to WHO preventive deworming recommendations, a biannual single-dose of albendazole (400 mg) or mebendazole (500 mg) is recommended as a public health intervention for all non-pregnant adolescent girls and women of reproductive age in order to reduce the worm burden of soil-transmitted helminthes which can affect nutritional status of adolescent girls [31]. In addition to this, a systematic review and meta-analysis indicated that taking deworming tablets improves the nutritional status of adolescent girls [32].

The educational status of the participant's fathers was significantly associated with the nutritional status of the study participants ($p<0.05$). Study participants whose fathers who had no formal education were 2.3 times more likely to be malnourished (thin) than those whose fathers completed college and university. This finding is in line with a study conducted in the cities of Tehran [33] and in Adama in Central Ethiopia [34].

Decision-making power for nutrition services was statistically associated with the nutritional status of the study participants ($p<0.001$). Adolescent girls from families whose decision-maker was the father or the mother were 1.9 and 2.02 times, respectively, more likely to be malnourished (thin) than adolescent girls who were from families in which both parents jointly make decisions for nutrition service.

Regularly skipping meals was significantly associated with the nutritional status of adolescent girls ($p<0.0001$). Adolescent girls who skip their regular meals were 2.8 times more likely to be malnourished than those who did not skipping their regular meals. This finding is supported by a study

conducted in Nigeria [35, 36] and in the Bale Zone [22]. This might be because skipping regular meals is an unhealthy eating behavior which can affect the nutritional status of adolescent girls [37].

The source of food was statistically associated with the nutritional status of adolescent girls (p<0.0001). Adolescent girls who were getting their food only from what their families produce or only purchasing it from the market were 3.28 and 5.14 times, respectively, more likely to be malnourished (thin) than those who were getting their food from both what their families produce and purchase from the market.

In addition to this, participants who were visited by a health extension worker at their home are more likely to be nutritionally normal. Visits by health extension workers were statistically associated with the nutritional status of adolescent girls (p<0.001). Adolescent girls who were not visited by health extension workers in their homes were 1.72 times more likely to be malnourished (thin) than those who were visited by health extension workers at their homes within the past three months. This might be due to nutritional counseling that can result in the improvement of nutritional knowledge and behavioral change for improved nutrition [38].

Factors associated with the nutritional status (HAZ) of adolescent girls in Southern Ethiopia

Decision-making power for nutrition services was statistically associated with HAZs of the study participants (p<0.05). Adolescent girls from families whose decision-maker the father or mother were 2.53 or 2.6 times, respectively, more likely to be malnourished(stunted) than adolescent girls from families which both parents jointly make decisions for nutrition services. Similarly, hand washing practices before eating and after using the toilet were statistically associated with the stunting of adolescent girls (p<0.05). Adolescent girls who did not wash their hands before eating and after using the toilet were 2.3 times more likely to be stunted than adolescent girls who were washing their hands before eating and after using the toilet. This is supported by a study conducted in Nepal [39]. Visits by health

extension workers in the community were statistically associated with the stunting status of adolescent girls ($p<0.05$). Adolescent girls who were not visited frequently by health extension workers at the community level were 2.04 times more likely to be stunted than those who were frequently visited by health extension workers at the community level.

Conclusions

Thinness and stunting are found to be high in the study area. Age, family size, monthly household income, regularly skipping meals, fathers' educational status, visits by health extension workers, and nutrition services decision-making power are the main predictors of thinness. Hand washing practice, visits by health extension workers, and nutrition services decision-making power are the main predictors of stunting among adolescent girls in Southern Ethiopia.

Recommendation

- At all levels, the girls' nutrition education/counseling should be given due emphasis as they are tomorrow's mothers which is very important to break the intergenerational cycle of malnutrition.
- Income-generating activities should be implemented to improve the monthly income status of the family as it affects the nutritional status of adolescent girls.
- Health extension workers should visit and give nutrition education regularly for adolescent girls at their homes and at community meetings.
- Hand washing practice should be improved before eating food and after using the toilet.
- Everything in the household should be decided jointly (both mother and father) to improve the ability to utilize resources among the household member.
- Health extension workers should give counseling for adolescent girls not to skip their regular meals.

Acknowledgments

Above all, I want to express my heartfelt thanks to the Almighty God, who helped me to accomplish this work and provides His blessing for me throughout my life. I acknowledge the Wolaita and Hadiya zones health office leaders and experts for their valuable cooperation during data collection and I would like to extend my gratitude to all the data collectors who participated in this research and adolescent girls who were willing to participate in this study. I am also grateful to the center of Food Science and Nutrition, Addis Ababa and Tufts University for facilitation and support.

Author Contributions

- Conceptualization: Yoseph Halala, Tefera Belachew, Cherinet Abuye, Abdulhalik Workicho, Kaleab Baye
- Data curation: Yoseph Halala, Tefera Belachew, Cherinet Abuye, Abdulhalik Workicho, Kaleab Baye
- Formal analysis: Yoseph Halala, Tefera Belachew, Cherinet Abuye, Abdulhalik Workicho, Kaleab Baye
- Funding acquisition: Yoseph Halala, Tefera Belachew, Cherinet Abuye, Abdulhalik Workicho, Kaleab Baye
- Investigation: Yoseph Halala, Tefera Belachew, Cherinet Abuye, Abdulhalik Workicho, Kaleab Baye
- Methodology: Yoseph Halala, Tefera Belachew, Cherinet Abuye, Abdulhalik Workicho, Kaleab Baye
- Project administration: Yoseph Halala, Tefera Belachew, Cherinet Abuye, Abdulhalik Workicho, Kaleab Baye
- Resources: Yoseph Halala, Tefera Belachew, Cherinet Abuye, Abdulhalik Workicho, Kaleab Baye
- Software: Yoseph Halala, Tefera Belachew, Cherinet Abuye, Abdulhalik Workicho, Kaleab Baye
- Supervision: Yoseph Halala, Tefera Belachew, Cherinet Abuye, Abdulhalik Workicho, Kaleab Baye

- Validation: Yoseph Halala, Tefera Belachew, Cherinet Abuye, Abdulhalik Workicho, Kaleab Baye
- Writing ± original draft: Yoseph Halala, Tefera Belachew, Cherinet Abuye, Abdulhalik Workicho, Kaleab Baye
- Writing ± review & editing: Yoseph Halala, Tefera Belachew, Cherinet Abuye, Abdulhalik Workicho, Kaleab Baye
- **Data Availability Statement:** All relevant data are within the paper and its Supporting information file. English Version Questionnaire and information sheet.docx.

- **Funding:** Addis Ababa University center for food science and nutrition and Wolaita Sodo University
- **Competing interests:** The authors have declared that no competing interests exist.

References

1. WHO, *Nutrition in adolescence: issues and challenges for the health sector: issues in adolescent health and development*. 2005: Geneva: World Health Organization.
2. CSA, *Ethiopia Demographic and Health Survey 2016.* Ababa, Ethiopia, and Rockville, Maryland, USA: CSA and ICF, 2016.
3. Ng, M., et al., *Global, regional, and national prevalence of overweight and obesity in children and adults during 1980–2013: a systematic analysis for the Global Burden of Disease Study 2013.* The lancet, 2014. **384**(9945): p. 766-781.
4. Mulugeta, A., et al., *Nutritional status of adolescent girls from rural communities of Tigray, Northern Ethiopia.* Ethiopian Journal of Health Development, 2009. **23**(1).
5. Demilew, Y.M. and A.A. Emiru, *Under nutrition and associated factors among school adolescents in Dangila Town, Northwest Ethiopia: a cross sectional study.* African health sciences, 2018. **18**(3): p. 756-766.
6. Martins, V.J., et al., *Long-lasting effects of undernutrition.* International journal of environmental research and public health, 2011. **8**(6): p. 1817-1846.
7. Black, R.E., S.S. Morris, and J. Bryce, *Where and why are 10 million children dying every year?* The lancet, 2003. **361**(9376): p. 2226-2234.
8. Guilloteau, P., et al., *Adverse effects of nutritional programming during prenatal and early postnatal life, some aspects of regulation and potential prevention and treatments.* J Physiol Pharmacol, 2009. **60**(Suppl 3): p. 17-35.
9. Melaku, Y.A., et al., *Prevalence and factors associated with stunting and thinness among adolescent students in Northern Ethiopia: a comparison to World Health Organization standards.* Archives of Public Health, 2015. **73**(1): p. 44.
10. Cnattingius, S. and E. Villamor, *Weight change between successive pregnancies and risks of stillbirth and infant mortality: a nationwide cohort study.* The Lancet, 2016. **387**(10018): p. 558-565.
11. Bay, J.L., S.M. Morton, and M.H. Vickers. *Realizing the potential of adolescence to prevent transgenerational conditioning of noncommunicable disease risk: multi-sectoral design frameworks*. in *Healthcare*. 2016. Multidisciplinary Digital Publishing Institute.
12. MoH, E.F., *Program Implementation Manual of National Nutrition Program (NNP)–I July 2008–June 2010.* Addis Ababa, Ethiopia: Federal Ministry of Health (MoH), 2008.

13. Nutrition, U.N.A.C.o.C.-o.S.-c.o. and I.F.P.R. Institute, *4th report on the world nutrition situation: nutrition throughout the life cycle*. 2000: United Nations, Administrative Committee on Coordination, Subcommittee on ….
14. Organization, W.H., *Adolescent nutrition: a review of the situation in selected South-East Asian countries*. 2006, WHO Regional Office for South-East Asia.
15. Bundy, D.A., et al., *Child and Adolescent Health and Development: Realizing Neglected Potential*, in *Child and Adolescent Health and Development. 3rd edition*. 2017, The International Bank for Reconstruction and Development/The World Bank.
16. Bekele, A., M. Kefale, and M. Tadesse, *Preliminary assessment of the implementation of the health services extension program: the case of southern Ethiopia.* Ethiop J Health Dev, 2008. **22**(3): p. 302-305.
17. Abrha, S., S. Shiferaw, and K.Y. Ahmed, *Overweight and obesity and its socio-demographic correlates among urban Ethiopian women: evidence from the 2011 EDHS.* BMC Public Health, 2016. **16**(1): p. 636.
18. Wassie, M.M., et al., *Predictors of nutritional status of Ethiopian adolescent girls: a community based cross sectional study.* BMC Nutrition, 2015. **1**(1): p. 20.
19. Gebregyorgis, T., T. Tadesse, and A. Atenafu, *Prevalence of thinness and stunting and associated factors among adolescent school girls in Adwa Town, North Ethiopia.* International journal of food science, 2016. **2016**.
20. Tamanna, S., et al., *Assessment of Nutritional Status among Adolescent Garo in Sherpur District, Bangladesh.* Bangladesh Journal of Medical Science, 2013. **12**(3): p. 269-275.
21. Awel, A.A., T.B. Lema, and H.J. Hebo, *Nutritional status and associated factors among primary school adolescents of pastoral and agro-pastoral communities, Mieso Woreda, Somali Region, Ethiopia: A comparative cross-sectional study.* Journal of Public Health and Epidemiology, 2016. **8**(11): p. 297-310.
22. Mohammed, A.Y. and T.B. Tefera, *Nutritional status and associated risk factors among adolescent girls in Agarfa high school, Bale Zone, Oromia Region, South East Ethiopia.* Int J Nutr Food Sci, 2015. **4**(4): p. 445-452.
23. Sibhatu, K.T. and M. Qaim, *Rural food security, subsistence agriculture, and seasonality.* PloS one, 2017. **12**(10): p. e0186406.
24. Amugsi, D.A., et al., *Women's participation in household decision-making and higher dietary diversity: findings from nationally representative data from Ghana.* Journal of Health, Population and Nutrition, 2016. **35**(1): p. 16.
25. Alenazi, S.A., et al., *Prevalence of obesity among male adolescents in Arar Saudi Arabia: future risk of cardiovascular disease.* Indian journal of community medicine: official publication of Indian Association of Preventive & Social Medicine, 2015. **40**(3): p. 182.
26. Adesina, A.F., et al., *Weight status of adolescents in secondary schools in port Harcourt using Body Mass Index (BMI).* Italian journal of pediatrics, 2012. **38**(1): p. 31.
27. Nurul, A., et al., *Nutritional status, dietary intake, and relevant knowledge of adolescent girls in rural Bangladesh.* Journal of Health, Population and Nutrition, 2010. **28**(1): p. 86-94.
28. Hossain, G.M., et al., *A study on nutritional status of the adolescent girls at Khagrachhari district in Chittagong hill tracts, Bangladesh.* American Journal of Life Sciences, 2013. **1**(6): p. 278-282.
29. Iyalomhe, S.I., et al., *Assessment of Dietary Habits and Nutritional Status of Adolescents in a Resource–Poor Environment in Nigeria.* International Journal of Nutrition and Food Sciences, 2018. **7**(4): p. 121.
30. Schuftan, C., *"Household purchasing-power deficit" A more operational indicator to express malnutrition.* Ecology of Food and Nutrition, 1979. **8**(1): p. 29-35.
31. Organization, W.H., *Guideline: preventive chemotherapy to control soil-transmitted helminth infections in at-risk population groups*. 2017: World Health Organization.
32. Ghogomu, E.T., et al., *Deworming in non-pregnant adolescent girls and adult women: a systematic review and meta-analysis.* Systematic reviews, 2018. **7**(1): p. 239.

33. Alavi, M., et al., *Dietary habits among adolescent girls and their association with parental educational levels.* Global journal of health science, 2013. **5**(5): p. 202.
34. Roba, K., M. Abdo, and T. Wakayo, *Nutritional status and its associated factors among school adolescent girls in Adama City, Central Ethiopia.* J Nutr Food Sci, 2016. **6**(493): p. 2.
35. Olumakaiye, M., T. Atinmo, and M. Olubayo-Fatiregun, *Food consumption patterns of Nigerian adolescents and effect on body weight.* Journal of nutrition education and behavior, 2010. **42**(3): p. 144-151.
36. Adesola, O.A., et al., *Breakfast habit and nutritional status of undergraduates in Ekiti State, Nigeria.* Sci J Public Health, 2014. **2**(4): p. 252-256.
37. Chin, Y. and M. Mohd Nasir, *Eating behaviors among female adolescents in Kuantan district, Pahang, Malaysia.* Pak J Nutr, 2009. **8**(4): p. 425-432.
38. Saibaba, A., et al., *Nutritional status of adolescent girls of urban slums and the impact of IEC on their nutritional knowledge and practices.* Indian J Community Med, 2002. **27**(4): p. 151-6.
39. Hamal, S., et al., *Prevalence of anaemia and its associated factors among school adolescent girls in Baglung municipality, Nepal.* Global journal of medicine and public health 2018. **7**(5).

Figure 1

Click here to access/download;Figure;Figures1.tif

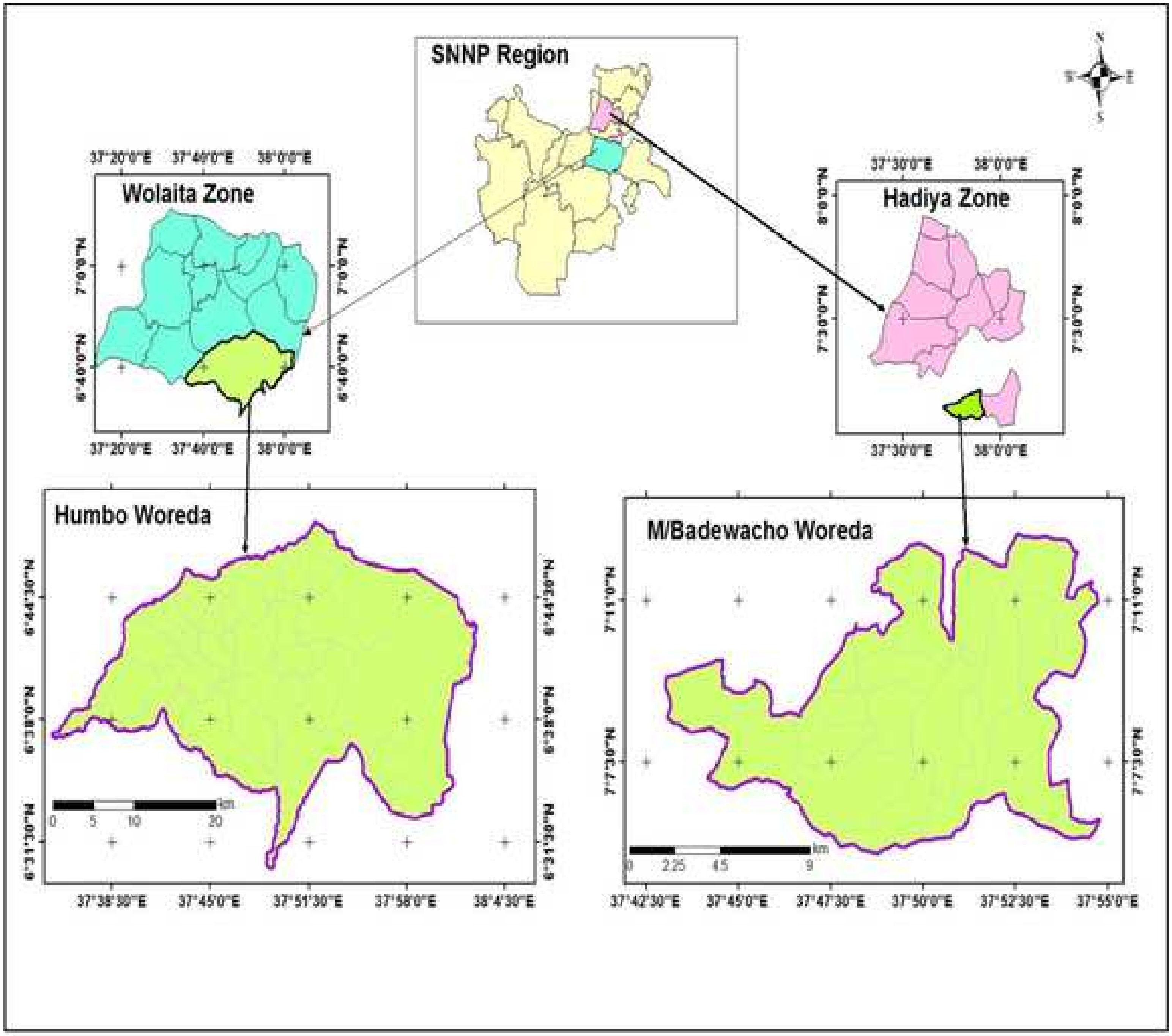

Figure1: Map of the study sites (Wolaita and Hadiya zones) in SNNPR, 2019

Figure2

Click here to access/download;Figure;Figures2.tif

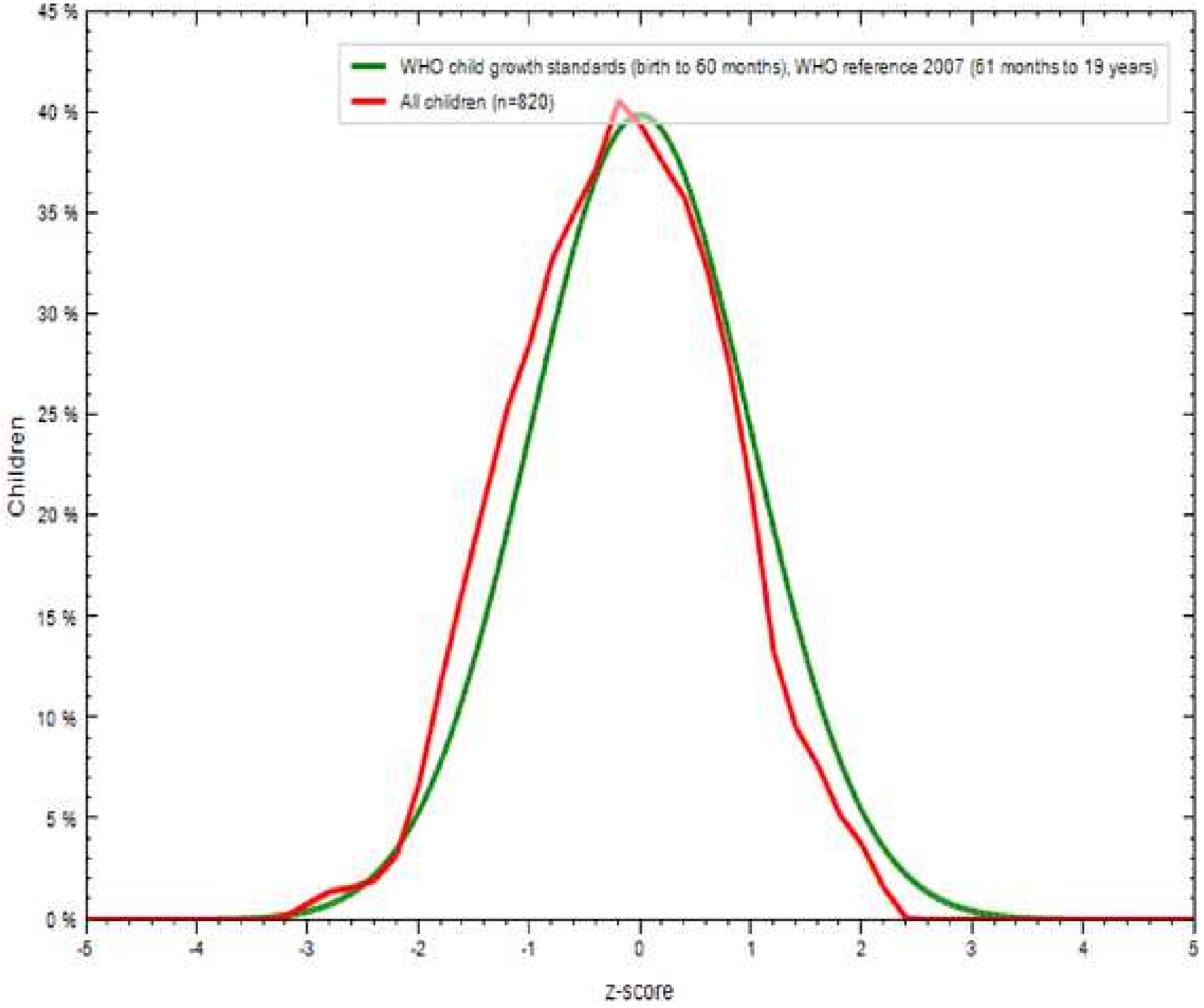

Figure2: Comparison of BMI-for-age (BAZ) of study population (N=820) with the 2007 WHO growth reference populations, 2019

Figure3

Click here to access/download;Figure;Figures3.tif

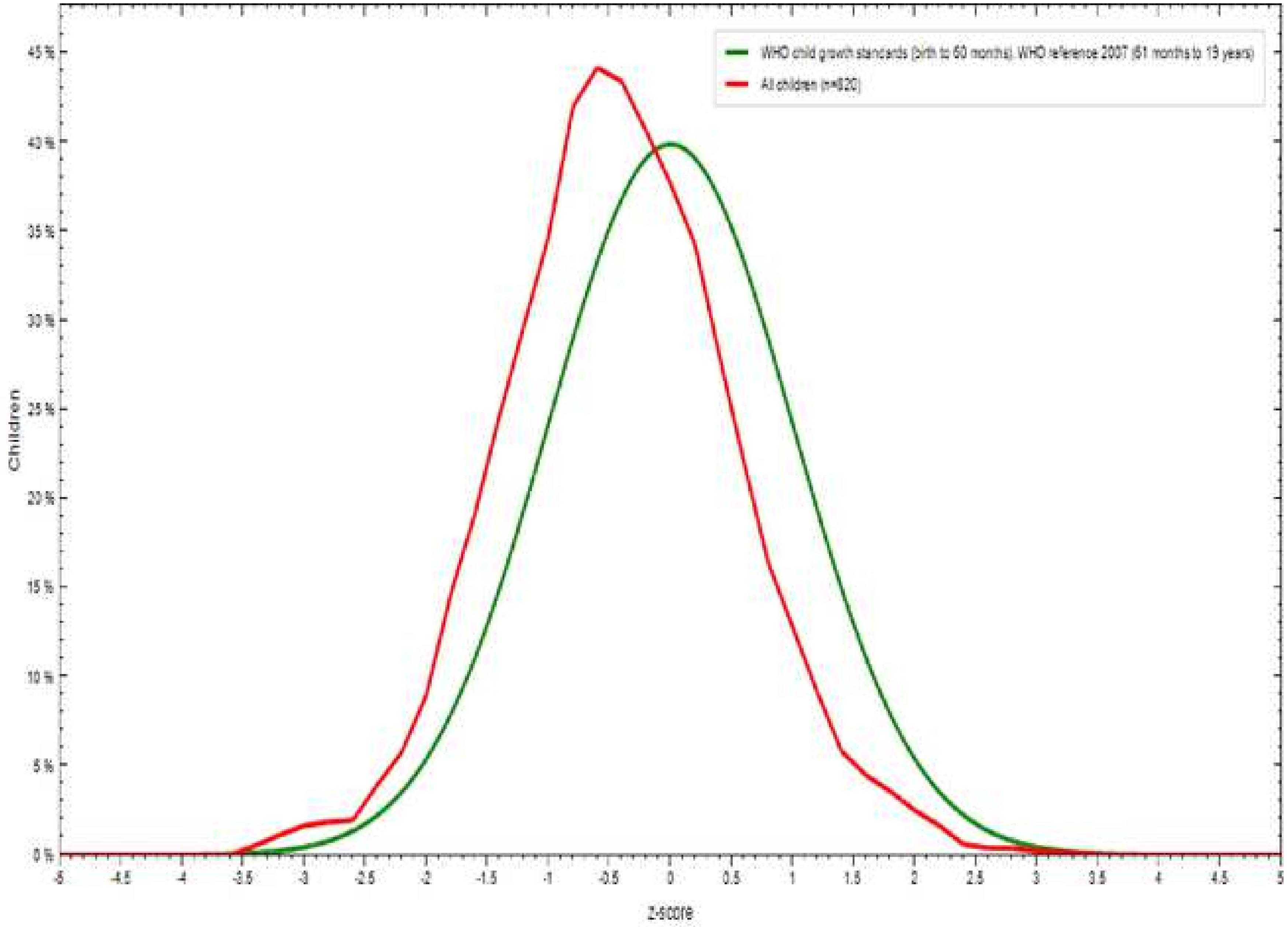

Figure3: Comparison of height-for-age (HAZ) of study population (N=820) with the 2007 WHO growth reference populations, 2019

Figure4

Click here to access/download;Figure;Figures4.tif

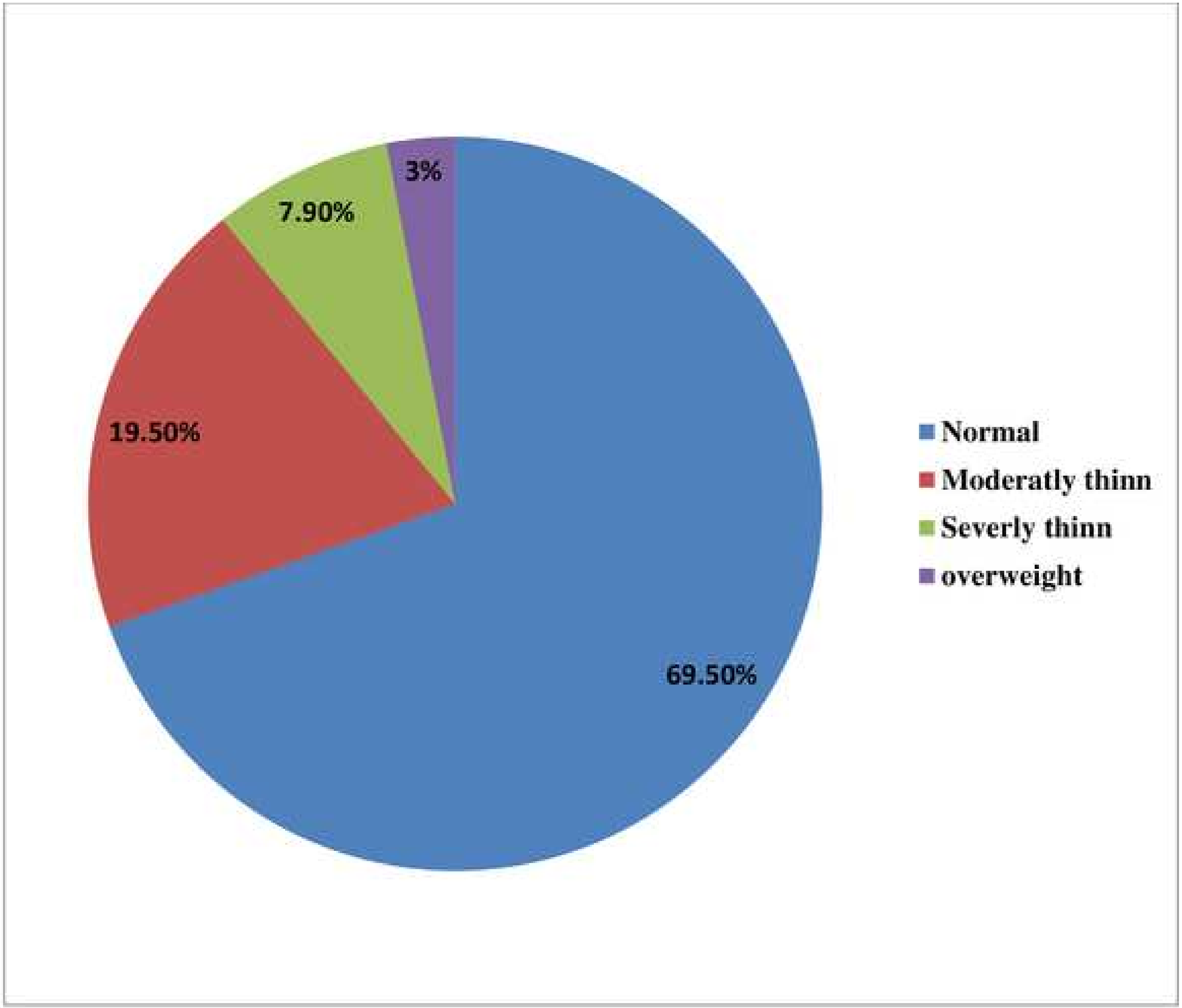

Figure4: Pi-chart representation of BMI for age Z-score (BAZ) among adolescent girls in southern Ethiopia, 2019.

Figure5 Click here to access/download;Figure;Figures5.tif

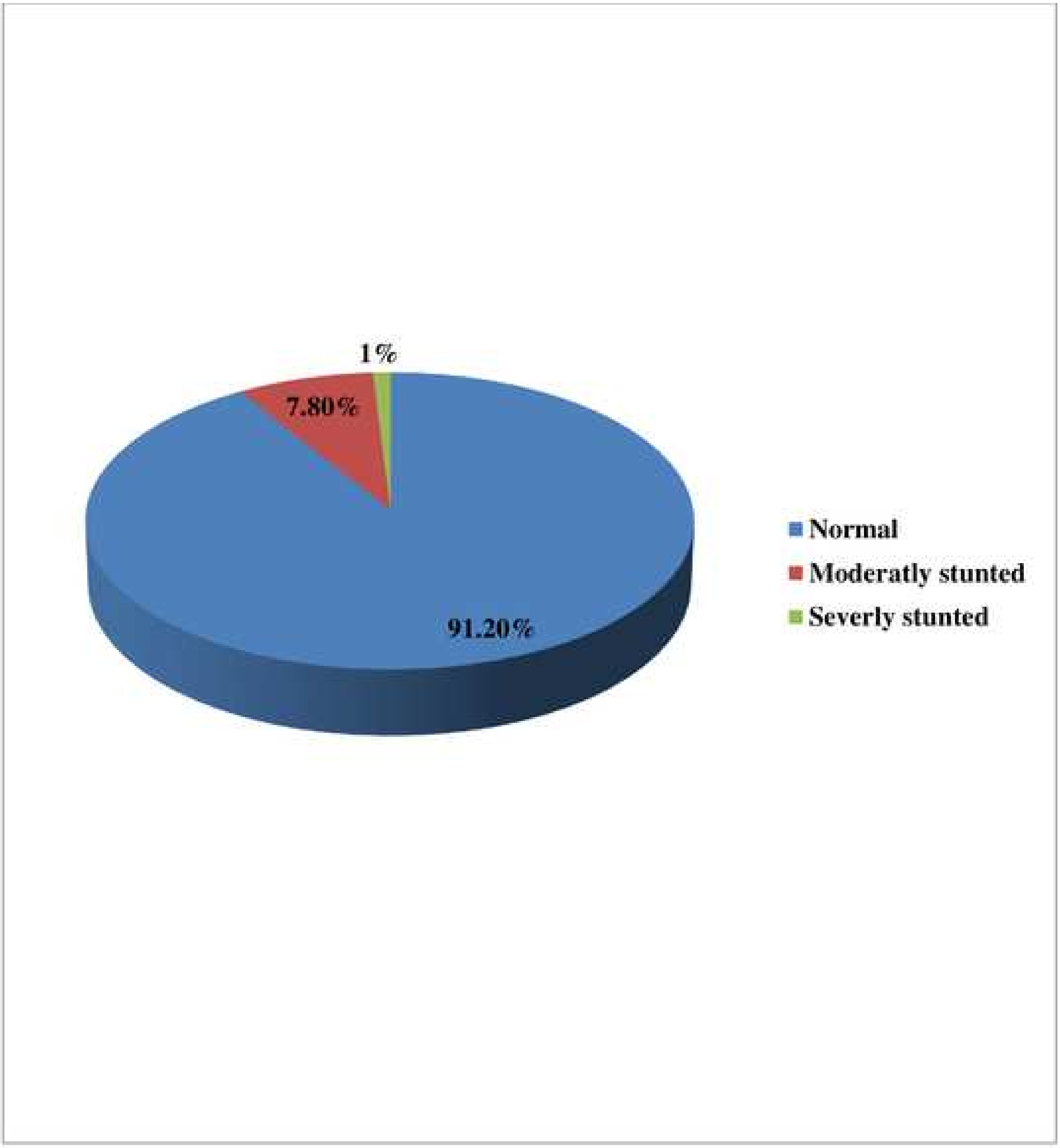

Figure5: Pi-chart representation of height for age Z-score (HAZ) among adolescent girls in southern Ethiopia, 2019

Track changed manuscript

Click here to access/download
Supporting Information
Manuscript2--Track changed.docx

Certificate of English editing

Click here to access/download
Supporting Information
Certificate_of_editing-ABPHH_2.pdf
