# Supplementary material for: Undernutrition and its determinants among adolescent girls in low land area of Southern Ethiopia
Source: PLoS One. 2021 Jan 12;16(1):e0240677. doi: 10.1371/journal.pone.0240677 (PMC7802945; doi:10.1371/journal.pone.0240677)
Supplement: Attachment — Submitted filename: Comments for authors.docx [file pone.0240677.s005.docx]

**REVIEWER’S COMMENTS TO THE AUTHORS**

**Abstract**

Line 7: multistage random sampling if the authors used random in all the stages.

Line 11: Did you actually estimate or generated through the software?

Line 12: Did you actually estimate or generated through the software?

Line 18: Let us know the nature of the association. This is not clear.

**Introduction**

Line 24: Defined by who?

**Materials and methods**

Line 61: Materials and methods please.

Line 66: Use an active verb such as shows instead of colon.

Line 71: BMI-for-age

Line 72: Height for age is a compound word and should be written as such.

Line 73: Add level

Line 78: You did not use both (thinness and stunting) for sample size determination.

Line 81: non response.

Line 88: simple random sampling method. Was it by balloting or what?

Line 92: Which type of random was used?

Line 93: Are kebele households? If no, how were households selected? There was no mention of house selection. What happened when there are more than one household per house?

Line 97: This is data collection method.

Line 100: How? Were the authors the ones that calibrated the scale? Is calibration the same as maintaining the scale at 0?

Line 101: Was this calibrated or did you attach a measuring tape to it? This is not clear.

Line 102: BMI-for-age

Line 102: Height-for-age

Line 102: It would be better if the method of calculating the Z scores is explicitly shown.

Line 104: Was anthropometry not part of your data collection method?

Line 106: B.Sc.

Line 107: experiences in collecting

Line 109: M.Sc.

Line 110: Data were

Line 122: rendered or translated

Line 125: This similar area needs to be mentioned.

Line 127: What was the reliability score?

Line 128: How many principal investigators did the study have?

Line 131: When did you standardize the measurements? In the field or during training? This is not clear.

Line 134: add (TEM)

Line 135: Let us have ENA in full first with the abbreviation in brackets.

Line 136: Were asked to instead of could.

Line 142: girls aged

**RESULTS:** Variables in the tables should be reported in the text as the appeared in the tables i.e. sequentially. Results must be reported in past tense.

Line 149: This figure was not shown in Table 1.

Line 150: This information is lacking in Table 1.

Line 150: This is not true of your Table 1.

Line 151: See Table 1 for clarity.

Line 151: Table 1 does not have 5-8 nor 1-4.

Line 153: This figure is not the same as the figure on Table 1.

Line 153: figures are not in the table.

Line 153: Do not use about when you are stating the exact figure.

Line 154: It is good to also include this value in USD.

Line 155: Let us have the value (>2000) in USD.

Line 156: Use colon and not period i.e. Table 1:

Table 1

Expunge level.

Line 172: Source: Field

Line 174: Expunge approximately because you are stating the exact figure.

Line 177: Who took

Line 177: 2 tablets of how many mg each?

Line 178: This is a bit confusing. Why would they take two tablets every 6 months?

Line 179: This is not clear. What do you actually mean by having supplement/receiving supplements? Is this the same as taking it? Be specific on the type of supplements. There are quite a number of them

Line 181: were not satisfied was not stated in Table 2. Either you include it in the table or you expunge it.

Line 185: Rearrange the variables so that they can be sequential. E.g. decision maker for nutrition service should be moved to after friendly nutrition service received.

On Table 2:

Expunge level.

Is nutrition education not part of friendly nutrition service?

When? i.e. received nutrition education i.e. how long ago?

Received deworming tablets: How long ago? Duration needs to be stated.

Friendly nutrition service given: use received instead of given.

Presence of cough, include 2 weeks before data collection.

Line 189: The table says of adolescent girls and not of the study participants.

Line 190: See Table 3 for the correct figure.

Line 190: girls lived in houses with mud floors

Line 191: study participants lived with domestic animals.

Line 194: Figure (90.1%) is not the same as what is in the table.

On Table 3:

Expunge level.

All sub percentages must add up to 100.0%

Recast as living with animals in the same house.

Number of windows in the entire house or per room? Be specific.

Questions are not used as variables. The total percent is not up to 100.0%. It is 98.4%

Line 201: as indicated in Table 4, about 39.5%........

Line 201: No, it does not show that 60.5% skipped meals because 4 and above is not the standard. We have 3 standard meals a day. It rather shows propensity to overweight due to overeating.

Line 206: 50.0% is not the same as the figure on the table.

Line 208: Meal pattern

Line 208: Why do you have a different table pattern here?

On Table 4

Expunge level.

Meal skipped: Percentage should be based on 496 as 100.0%.

Line 212: I believe that you mean -1 and median. Normal includes this and +1. So normal BMI-for-age refers to Z scores between -2 and +2 (excluding -2 and +2) or you say it ranges from -1 to +1.

Line 213: Moderate thinness or moderate stunting is BMI-for-age or Height-for-age Z score that equals -2 or you say less than (<) -1.

Line 214: Severe thinness or severe stunting is BMI-for-age or height-for-age Z score <2 or exactly

-3.

Line 217 - 220: The figures should come in after Table 5.

Line 225: Details of the associations should be stated in all cases e.g. were the younger adolescents affected more than the older ones? Were children from households with family size of >5 affected more than those with 5 and less?

Line 228: The variables should be discussed sequentially as they appeared in all the tables.

Line 232: The use of frequencies did not equalize the figures for comparison. I suggest the authors translate the N values to percentages for easy comparison.

On Table 6: BAZ is ≤-2

Line 234: Is this right? Do you not mean <0.01 for 0.001 and <0.001 for 0.000?

Discussion

Line 246: Restatement of results are not allowed. You can only refer to them.

Line 252: than the findings/results from a study......

Line 253: observed undernutrition among the……..

Line 254 to 259 are on results. Results are not restated in discussion.

Line 260: indicated that hand washing with soap after using the toilet and before eating affects the nutritional status of adolescent girls. How? This should be made clear.

Line 261-262: This might lead to the low nutritional status of adolescent girls. Explain the mechanism through which this occurs.

Line 262: the result of a study conducted………

Line 264: replace was with as.

Line 266: How does it affect their nutritional status?

Line 269-271: Please state the implications of the results stated.

Line 275 and throughout the discussion session, expunge malnourished and use only the specific terms: thinness or stunting.

Line 275: This is the association I expected to see in the result section. Please move it there and explain all associations there.

Line 290-292: According to WHO preventive deworming recommendations, a biannual single-dose of albendazole (400 mg) or mebendazole (500 mg) is recommended. If this is so, why did you have 2 tablets?

Line 293-294: What is the mechanism of action?

Lines 302-309: What you have here is result and not discussion.

Line 309: expunge malnourished and use thin.

Lines 324 to 331 were on results. Tell us the implications of your findings.

Line 325: ‘whose decision-maker the father or mother’ what is missing here?

Acknowledgement

The use on ‘I’ indicates single author.
