# Supplementary material for: Undernutrition and its determinants among adolescent girls in low land area of Southern Ethiopia
Source: PLoS One. 2021 Jan 12;16(1):e0240677. doi: 10.1371/journal.pone.0240677 (PMC7802945; doi:10.1371/journal.pone.0240677)
Supplement: Attachment — Submitted filename: Response to Reviewers.docx [file pone.0240677.s006.docx]

First of all, I would like to thank academic editor and reviewer#1 for their scientific, reasonable and convincing comments and questions. Really, I have got interesting lesson from them. Thank you very much.

I tarried to respond to all comments and questions bellow:

**Response for comments and questions raised by academic editor**

1. We ensure that our manuscript meets PLOS ONE's style requirements. Our manuscript was edited and formatted by ‘editage Cactus’-licensed English edition.
2. We have developed questionnaire after reading different literature based on objective of our study. We attached the questionnaire as supporting documents during manuscript submission.
3. Yes, I have ORCID iD: <https://orcid.org/0000-0001-6191-9027>
4. We have referred to figure 1-5 in our text
5. All figures were not copied from other persons’ work. There are no copyright holders for these figures. These all figures are my own work. I have developed/ drawn these figures by using WHO Anthro plus, SPSS and ArcGIS software.

**Response for reviewer#1 comments and questions**

**Abstract**

Line 7: multistage random sampling if the authors used random in all the stages.

Response: Yes, random sampling procedure was used. Equal chance was given all participants.

Line 11: Did you actually estimate or generated through the software?

Response: we generated through software by using SPSS version 21.0.

Line 12: Did you actually estimate or generated through the software?

Response: we generated through software by using SPSS version 21.0.

Line 18: Let us know the nature of the association. This is not clear.

Response: Nature of association is positive.

Large family size, low monthly income , not taking deworming tablets , low educational status the fathers’, the source of food for the family only from market, not visited by health extension workers and not washing hand before eating and after using the toilet were positively associated with undernutrition of adolescent girls.

**Introduction**

Line 24: Defined by who?

Response: age range of adolescent was defined by world health organization (WHO).

**Materials and methods**

Line 61: Materials and methods please.

Response: we changed it to “Materials and methods”

Line 66: Use an active verb such as shows instead of colon.

Response: we changed it to Figure 1 shows

Line 71: BMI-for-age

Response: we changed it to BMI-for-age

Line 72: Height for age is a compound word and should be written as such.

Response: we changed it to height-for-age

Line 73: Add level

Response: we added “level”

Line 78: You did not use both (thinness and stunting) for sample size determination.

Response: we used both thinness and stunting for sample size determination. But, we took stunting because it gave larger sample size.

Line 81: non response.

Response: we changed it to response

Line 88: simple random sampling method. Was it by balloting or what?

Response: we inserted “sampling”; we used SPSS version 21.0 software for random selection. When we ran, it automatically selected 843 adolescent girls from all listed adolescent girls. First census was developed by health extension girls from each selected kebele.

Line 92: Which type of random was used?

Response: If there was more than one adolescent girl in a household, one adolescent girl was randomly selected by lottery method.

Line 93: Are kebele households? If no, how were households selected? There was no mention of house selection. What happened when there are more than one household per house?

Response: No, kebeles are not households. Kebeles are “the lowest administrative unit in Ethiopia.”

Kebele includes many households. In average, 500-800 households are in one kebele. Census of adolescent girls was conducted from all randomly selected kebeles. So, all households in the randomly kebele which were with eligible adolescent girls were included in census.

Line 97: This is data collection method.

Response: we corrected it.

Line 100: How? Were the authors the ones that calibrated the scale? Is calibration the same as maintaining the scale at 0?

Response: -No, the authors are not the ones that calibrate the scale. Data collectors calibrate the	scale at each time whether it works properly or not.

- Calibration is not only maintaining the scale at 0, but also it includes checking the tool is working correctly.

Line 101: Was this calibrated or did you attach a measuring tape to it? This is not clear.

Response: we used the height measuring board which was with measuring tape. The measuring tape was attached to the board and fixed already.

Line 102: BMI-for-age

Response: we corrected it

Line 102: Height-for-age

Response: we corrected it

Line 102: It would be better if the method of calculating the Z scores is explicitly shown.

Response: we used WHO Anthro-plus software to calculate Z- score from weight, height and age in month of adolescent girls.

Line 104: Was anthropometry not part of your data collection method?

Response: anthropometry is parts of data collection method. We corrected it

Line 106: B.Sc.

Response: we corrected it

Line 107: experiences in collecting

Response: we corrected it

Line 109: M.Sc.

Response: we corrected it

Line 110: Data were

Response: we corrected it

Line 122: rendered or translated

Response: Back translation to English was conducted

Line 125: This similar area needs to be mentioned.

Response: In similar kebeles which were selected for the study.

Line 128: How many principal investigators did the study have?

Response: one, we corrected it.

Line 131: When did you standardize the measurements? In the field or during training? This is not clear.

Response: Standardization was took place during training

Line 134: add (TEM)

Response: we added (TEM)

Line 135: Let us have ENA in full first with the abbreviation in brackets.

Response: Emergency Nutrition Assessment (ENA)

Line 136: Were asked to instead of could.

Response: it was corrected

Line 142: girls aged

Response: it was corrected

**RESULTS:** Variables in the tables should be reported in the text as the appeared in the tables i.e. sequentially. Results must be reported in past tense.

Line 149: This figure was not shown in Table 1.

Response: we inserted/corrected the missed figure

Line 150: This information is lacking in Table 1.

Response: we inserted/corrected the lacking information

Line 150: This is not true of your Table 1.

Response: It was corrected

Line 151: See Table 1 for clarity.

Response: It was corrected

Line 151: Table 1 does not have 5-8 nor 1-4.

Response: It was corrected

Line 153: This figure is not the same as the figure on Table 1.

Response: It was corrected

Line 153: figures are not in the table.

Response: It was corrected

Line 153: Do not use about when you are stating the exact figure.

Response: It was corrected

Line 154: It is good to also include this value in USD.

Response: <31.25 USD

Line 155: Let us have the value (>2000) in USD.

Response: >62.5 USD

Line 156: Use colon and not period i.e. Table 1:

Table 1

Response: we used colon

Expunge level.

Response: Level was expunged

Line 172: Source: Field

Response: It was corrected

Line 174: Expunge approximately because you are stating the exact figure.

Response: the word approximately was expunged

Line 177: Who took

Response: it was corrected

Line 177: 2 tablets of how many mg each?

Response: At this research study area, albendazole (400 mg) was given every six months for adolescent girls. But, adolescent girls don’t know the dose and types of deworming tablets. During data collection period, data collectors asked whether adolescent girls took deworming tablets or not. If they took, how many (one or two) deworming tablet. In the study area some adolescent girls were no taking deworming tablets. Because, they don’t know the importance of it.

Line 178: This is a bit confusing. Why would they take two tablets every 6 months?

Response: in Ethiopia, adolescents were universally supplemented deworming tablets every six month. That means, twice annually adolescent girls were taking deworming tablets

Line 179: This is not clear. What do you actually mean by having supplement/receiving supplements? Is this the same as taking it? Be specific on the type of supplements. There are quite a number of them

Response: that means taking iron-folic acid tablet

Line 181: were not satisfied was not stated in Table 2. Either you include it in the table or you expunge it.

Response: It was expunged

Line 185: Rearrange the variables so that they can be sequential. E.g. decision maker for nutrition service should be moved to after friendly nutrition service received.

On Table 2:

Response: it was corrected

Expunge level. It was corrected. It was removed

Is nutrition education not part of friendly nutrition service?

Response: friendly nutrition education is parts of nutrition education.

It is how they are giving nutrition education politely. Friendly nutrition education is the way of approach how health extension giving nutrition education for adolescent girls.

When? i.e. received nutrition education i.e. how long ago?

Response: with in the last three months

Received deworming tablets: How long ago? Duration needs to be stated.

Response: twice annually deworming tablets were given for adolescent girls.

Friendly nutrition service given: use received instead of given.

Response: It was corrected

Presence of cough, include 2 weeks before data collection.

Response: All are corrected by track change

Line 189: The table says of adolescent girls and not of the study participants.

Response: corrected as ‘’adolescent girls’’

Line 190: See Table 3 for the correct figure.

Response: It was corrected

Line 190: girls lived in houses with mud floors

Response: it was corrected

Line 191: study participants lived with domestic animals.

Response: it was corrected

Line 194: Figure (90.1%) is not the same as what is in the table.

On Table 3:

Response: it was corrected as ‘’93.2%’’

Expunge level.

Response: level expunged

All sub percentages must add up to 100.0%

Response: it was corrected

Recast as living with animals in the same house.

Response: it was corrected

Number of windows in the entire house or per room? Be specific.

Response: It is the number of windows in the entire house

Questions are not used as variables.

Response: we have changed questions to variables

The total percent is not up to 100.0%. It is 98.4%

Response: It was corrected

Line 201: as indicated in Table 4, about 39.5%........

Response: It was corrected

Line 201: No, it does not show that 60.5% skipped meals because 4 and above is not the standard. We have 3 standard meals a day. It rather shows propensity to overweight due to overeating.

Response: we corrected it. The right comment was given for us. We give many thanks for the reviewer#1.

Line 206: 50.0% is not the same as the figure on the table.

Response: It was corrected. It is 50.6 % on the table

Line 208: Meal pattern

Response: It was corrected

Line 208: Why do you have a different table pattern here?

On Table 4

Expunge level.

Response: level expunged

Meal skipped: Percentage should be based on 496 as 100.0%.

Response: It was corrected i.e. removed from the table

Line 212: I believe that you mean -1 and median. Normal includes this and +1. So normal BMI-for-age refers to Z scores between -2 and +2 (excluding -2 and +2) or you say it ranges from -1 to +1.

Response: We used WHO 2010 manual for comparison and We used WHO Anthro plus software for Z-score calculation.

For this study: Normal, -2 < BAZ< +2

Moderately thin, -3 ≤ BAZ < -2

Severely thin, BAZ < -3

Overweight, BAZ ≥+2[. \anthro_pc_manual_v322.pdf](../anthro_pc_manual_v322.pdf)

Line 213: Moderate thinness or moderate stunting is BMI-for-age or Height-for-age Z score that equals -2 or you say less than (<) -1.

Response: Moderately thin, -3 ≤ BAZ < -2 or moderately stunted -3 ≤ HAZ < -2

Line 214: Severe thinness or severe stunting is BMI-for-age or height-for-age Z score <2 or exactly

-3.

Response: Z-score is < -3.

Line 217 - 220: The figures should come in after Table 5.

Response: Now, I put figures after Table 5.

Line 225: Details of the associations should be stated in all cases e.g. were the younger adolescents affected more than the older ones? Were children from households with family size of >5 affected more than those with 5 and less?

Response: the details of the associations were now stated well.

Line 228: The variables should be discussed sequentially as they appeared in all the tables.

Response: It was corrected

Line 232: The use of frequencies did not equalize the figures for comparison. I suggest the authors translate the N values to percentages for easy comparison.

Response: we translated frequencies to percentages

On Table 6: BAZ is ≤-2

Response: It was corrected as BAZ is ≤-2

Line 234: Is this right? Do you not mean <0.01 for 0.001 and <0.001 for 0.000?

Response: it was corrected. We mean <0.01 for 0.001 and <0.001 for 0.000. Thank you very much

**Discussion**

Line 246: Restatement of results are not allowed. You can only refer to them.

Response: we corrected it

Line 252: than the findings/results from a study......

Response: we corrected it

Line 253: observed undernutrition among the……..

Response: we corrected it

Line 254 to 259 are on results. Results are not restated in discussion.

Response: now we have written it well.

Line 260: indicated that hand washing with soap after using the toilet and before eating affects the nutritional status of adolescent girls. How? This should be made clear.

Response: This might be due to adolescent girls who were not washing their hand before eating and after using toilet can be affected by diarrhea and other communicable diseases. If they affected by diarrhea and other communicable diseases, absorption as well as utilization of nutrient can be affected, and which can lead to stunting.

Line 261-262: This might lead to the low nutritional status of adolescent girls. Explain the mechanism through which this occurs.

This might be due to adolescent girls who were not washing their hand before eating and after using toilet can be affected by diarrhea and other diseases and which can lead to stunting.

Line 262: the result of a study conducted………

Response: It was corrected

Line 264: replace was with as.

Response: It was corrected

Line 266: How does it affect their nutritional status?

Response: Buying food from distant market takes time and which can affect accessibility of the household for the food consumption. Sometimes market price fluctuation can affect food consumption. So, this might affect nutritional status of the adolescent girls.

Line 269-271: Please state the implications of the results stated.

As indicated by the study conducted in Jimma Zone and World Health Organization report, women autonomy in decision-making is important to improve nutritional status themselves and their children.

Line 275 and throughout the discussion session, expunge malnourished and use only the specific terms: thinness or stunting.

Response: we expunged malnutrition and used specific term thinness or stunting

Line 275: This is the association I expected to see in the result section. Please move it there and explain all associations there.

Response: now we corrected it well.

Line 290-292: According to WHO preventive deworming recommendations, a biannual single-dose of albendazole (400 mg) or mebendazole (500 mg) is recommended. If this is so, why did you have 2 tablets?

Response: At this research study area, albendazole (400 mg) was given every six months for adolescent girls. But, adolescent girls don’t know the dose and types of deworming tablets. During data collection period, data collectors asked whether adolescent girls took deworming tablets or not. If they took, how many (one or two) deworming tablet. In the study area some adolescent girls were no taking deworming tablets. Because, they don’t know the importance of it.

Line 293-294: What is the mechanism of action?

Response: Intestinal parasite may share the food that adolescent girls have eaten. Sometimes, intestinal parasite cause diarrhea disease and which can decrease absorption of important nutrients. This can lead to low nutritional status of adolescent girls.

Lines 302-309: What you have here is result and not discussion.

Response: It was corrected

Line 309: expunge malnourished and use thin.

Response: it was corrected. Malnourished was changed to specific word “thin”

Lines 324 to 331 were on results. Tell us the implications of your findings.

Response: now the implications are written well

Line 325: ‘whose decision-maker the father or mother’ what is missing here?

Response: here is ‘’either…or’’ missing. we have corrected it.

**Acknowledgement**

The use on ‘I’ indicates single author.

Response: it was corrected as “we”

**Responses to the comments raised by Reviewer #1**

i. there is no specific research hypotheses that are being examined or tested;

Response: there are specific research hypothesis that are stated well at the end of introduction section.

Research hypothese1. Prevalence of under nutrition among adolescent girls in the study area is high

Research hypothesis2. Socio-economic, nutrition service, health and hygiene related variable are associated factors for under nutrition among adolescent girls

ii. there are several other similar published papers from Ethiopia examining factors associated with undernutrition and stunting in adolescent girls, so it is not apparent that this study adds any new or significant insights (see references number 18, 19, 21, 22)-other than being in a different region of the country;

Response: study is new for southern Ethiopia among adolescent girls.

iii. this study does not include any novel approaches or methods that bring original or new insights into tackling nutritional challenges in the population;

Response: our study design is community based cross sectional, and which give equal chance to all adolescent girls to be included in the study. Most of the research conducted on school adolescent girls and which cannot be generalized for adolescent girls who are not following the school. But our research based on community.

iv. the study does not consider the changes in BMI and height during the adolescent growth	spurt and the timing of this relative to the growth spurt of the reference population used	to calculate z-scores. Age should be included as a continuous variable in analyses to try	to lesson the effects of age differences in adolescent growth;

**Response:** Age of the adolescent girls grouped as 1) Early adolescent (10-13years), 2)	middle adolescents (14-16 years) and 3) late adolescents (17-19 years). WHO as well as	many literatures grouped adolescent girls’ age like this. Growth spurt of adolescent girls is	different in different age groups. Growth spurt of adolescent girls is high during 10-13 years old than 17-19years. During 10-13 years age, they need high quality and quantity diet. Analyzing age as continuous variable by linear regression model and analyzing age as grouped variable by binary logistic regression, there are no the effects of age differences in adolescent growth. So, our analysis by grouping age is not wrong.

v) a whole range of variables are analyzed without a specific rationale of how these relate to the research hypotheses.

Response: variables were analyzed with the specific rationale in relation to research	hypotheses.

Vi) Developing a conceptual framework based on detailed review

I developed conceptual framework from literature review. Most of the time used for analysis but not submitted with manuscript.

Socio demographic factors

- Educational status

of girls

- Occupation of parents
- Religion
- Family size
- Age
- Monthly income
- Educational status of parents

Health and sanitation related factors

- Malaria illness
- Cough
- Taking IFAS
- Taking deworming
- Nutrition education
- Alcohol drinking
- smoking,
- chat chewing,
- presence or absence of latrine
- Source of drinking water.
- Hand washing practice
- Drinking water
- Types of toilet
- Dirrharia illness

UNDER NUTRITION

(Low BAZ &HAZ)

Dietary related factors

- Type of diet
- meal frequency
- skipping meal
- Source of food

Figure 1Conceptual Frame work for low nutritional status: Reviewed from literatures
