# Supplementary material for: Undernutrition and its determinants among adolescent girls in low land area of Southern Ethiopia
Source: PLoS One. 2021 Jan 12;16(1):e0240677. doi: 10.1371/journal.pone.0240677 (PMC7802945; doi:10.1371/journal.pone.0240677)
Supplement: Attachment — Submitted filename: Response to Reviewers2nd.docx [file pone.0240677.s007.docx]

First of all, I would like to thank academic editor and Reviewer #2 and Reviewer #3 for their scientific, reasonable and convincing comments and questions. Really, we have got interesting lesson from them. Thank you very much again!

I tarried to respond to all comments and questions bellow:

**Response for comments and questions raised by academic editor**

1. Our manuscript was edited and formatted by ‘editage Cactus’-licensed English edition.

If additional English edition is needed, they will edit our manuscript again.

2. We have listed adolescent girls from selected kebeles (villages) and created sampling frame (listing of adolescent girls in the selected kebeles). From this sampling frame simple random sampling procedure was applied to select 843 participants.
3. We ensure that all the reviewers’ comments were well addressed. We are ready to respond to any comments. Thank you very much!

**Response for reviewer#2 comments and questions**

**Abstract**

Line 10: Replace estimated with generated.

Response: It was corrected. Generated replaced estimated

Line 17: Please add poor to nutritional status.

Response: It was corrected. The word ‘Poor’ added to nutritional status.

**Introduction**

Line 23: Please reference WHO properly with the appropriate number.

Response: It was corrected
Materials and methods
Line 93: was selected by simple random sampling (lottery method).

Response: corrected. Was selected by simple random sampling(lottery method).

Line 108: B.Sc. Please add period after c.

Response: Corrected. Period was added after c
Line 130: investigator

Response: Corrected
Results
Line 156: Please do not expunge your currency. Use your currency but insert USD in parenthesis for easy estimation of value e.g. > 2000 ETBirr (62.5 USD)

Response: Corrected as example was given.
Table 1: Variables not variable

Response: It was corrected as Variables
Table 1: Please see earlier comment. Let the USD value be in brackets. An example has been given.

Response: It was corrected
N.B. All results must be presented in past tense.

Response: It was corrected. Now all results are presented in past tense.
See the example below:
Line 216 to 217: Report all results in past tense
E.g.
1. 48.7% of the study participants brushed their teeth.....
2. 53.3% washed........
3. 41.7% usually washed their........
4. 3.4% did not wash....... etc
Please results should be reported in past tenses.

Response: It was corrected. All were written by past tense
Lines 233 and 238: Do Table 5 and figures 4 and 5 have the same information? I think the information they contain should be described differently.

Response: off course they have the same information. But we added the figures 4 and 5	to elaborate information more.
Line 250: Low BAZ was statistically….

Response: It was corrected
Table 6
Total for age vs BAZ ≤ -2 =227; BAZ > -2 = 587 = 814. What happened to 6 respondents?

Response: It was corrected. 6 respondents were in BAZ > -2. It was typing error.
Please do not expunge your currency. Use your currency but insert USD in parenthesis for easy estimation of value e.g. > 2000 ETBirr (62.5 USD)

Response: It was corrected. We added our currency.

Line 258: height-for-age

It was corrected
Line 259: Add low to HAZ of the study participants were statistically….

Response: It was corrected. Low added to HAZ
Table 7
Expunge level

Response: It was corrected. Level expunged from the table.

HAZ ≤ -2 and not <-2
HAZ ≤ -2 =72 HAZ > -2 = 748 but Visited by a health extension worker has HAZ ≤ -2 =74 HAZ > -2 = 746. Which one is correct?

Response: HAZ ≤ -2 =72 HAZ > -2 = 748 is correct.
Discussion
Line 277: which can lead to diarrhoea.
Lines 304-305: Please do not expunge your currency. Use your currency but insert USD in parenthesis for easy estimation of value e.g. > 2000 ETBirr (62.5 USD).

Response: It was corrected. Our currency was added and USD is in parenthesis
Line 308: Families with lower monthly incomes are more likely to eat

Response: It was corrected
Line 309: expunge that
Response: It was corrected. That was expunged
N.B. I am not at peace with the tenses used in the result and discussion sections. The tenses should be reviewed. This is very important.

Response: Now we have corrected grammatical and spelling error. Previously it was edited by ‘editage Cactus’-licensed English edition. If again additional English edition needed, they are volunteer to edit again.

Thank you very much for your comments!

**Response for reviewer#3 comments and questions**

Abstract
1. Line 14, should read low educational status of father, not status the father’s

Response: It was corrected. It was changed to low educational status of father
2. This is a structured abstract, therefore I entreat the authors to separate their	conclusion from the results section in the abstract

Response: we separated them.
3. If the authors wish to caption the background of the abstract as background, then the introduction as found in the main manuscript must be changed to background or vice versa for consistency sake

Response: It was corrected. ‘Introduction’ in main manuscript was changed to ‘background’
Background
4. Replace developing countries with low and middle-income countries, line 25 page 1

Response: developing countries was replaced with low and middle-income countries.
5. Line 34 there is no full stop

Response: we added full stop.
6. Line 46, page 2, how large is large. Specify the exact proportion or percentage

Response: magnitude of undernourished were described on the manuscript.
7. Line 55 page 2, the sentence…is missing a connection. It presupposes the authors have reviewed previous studies conducted in the study area or creating a gap. As this is a great step, the gap is not clear/well-articulated. Please the gap very clear and strong.

Response: It was corrected well.
8. The literature review on the predictors of under nutrition is not adequate. This needs strengthening. In addition, provide specific references for particular predictors. E.g low household income [2], age [3], type of place of residence [4] etc…

Response: Now it was corrected well

9. In addition, the predictors the authors found in their study were not reviewed at the background to inform the discussion appropriately

Response: Now it was corrected well. Predictors of undernutrition were reviewed and written at the background.

Materials and methods
10. Between line 60 and 61, please provide a sub-section “study area/setting”

Response: It was corrected. Sub-section “study area’’ was added
11. Line 79 check the statement. It doesn’t read well recast

Response: It was corrected
Sampling procedure
12. Line 87, p3 what informed the selection of the 2 zones? Likewise the selection of the of the three kebeles why 3?

Response: 2 zones were selected purposively, because there is no study conducted in the area. But the selection of 3 kebeles (village) was random by considering 30% of total population principle.

13. Line 92, the authors stated that they used a census, why then did they use a lottery method to select one adolescent in household with more than one adolescent?

Response: If there was more than one adolescent girl in a household, one adolescent girl was selected by simple random. Because to get heterogeneity of the study participants by socio-economic factors.

14. Again, if the authors claimed they used a census, why did they have a sample size?

Response: Sorry for inappropriate word ‘census’. We mean listing of adolescent was taken place before sampling. After completing listing of adolescent from all selected villages (kebeles), 820 adolescent girls were selected from all listed adolescent girls by simple random sampling method.
15. Apart, from the issues raised, with regards to the census, the sampling approach the authors used is not well-articulated. This should be explained in detail.

Response: Listing of adolescent girls from each village/kebele was conducted to create sampling frame. Creating sampling frame is very important for simple random sampling method.

16. Line 67-68, the authors mentioned that both in and out of school adolescents constituted the study sample. What was then the exclusion criteria? See line 96-97. Please describe those who were not eligible.

Response: Adolescent girls with pregnancy, physical and mental disability were excluded from the study.

Data collection
17. Line 107 kindly make reference to the literature you adapted instrument/questionnaire from

Response: It was corrected. References cited well
18. Also make reference that the instrument is attached as a supplementary file.

Response: It was corrected. References cited well
19. Describe how the independent variables were measured. Especially monthly income. Why didn’t the authors use the approach by the demographic and health survey which uses household assets to create the wealth variables by using the principal component analysis technique

Response: We were not interested to know wealth status of the household. But we were interested to know how much monthly they earn and how this is related with nutritional status of the adolescent girls.

Sometimes household assets are not guaranty for nutritional status. Even cash crop production and edible crop production of the household have different nutritional status implication. Thus, we used monthly income only.

Data quality assurance
20. Specify the area the pretesting was done

Response: Study was conducted at Agodama, Ampo koysha, Gututolarena, Kachera, Mazoriya and Woira kebeles(villages). Pre-testing was conducted at Sipa and shone kebeles(villages)which have similar background.
Results
21. Check Table 1, the column with the percentages are not well presented

Response: It was corrected
Discussion
22. Line 268, where is the reference for the study you are comparing your results with?

Response: It was corrected. Reference was added/cited

23. Line 276-277, please recast

Response: It was corrected

24. Line 291, the independent variables should come before the outcome variable.

Response: It was corrected

25. The authors failed to acknowledge the strength and weaknesses in their study. This should be discussed extensively

Response: Now strength and weaknesses of the study were written very well.
26. At the acknowledgement section, it appears the study was conducted by one author. This should be relooked at.

Response: It was corrected

27. The authors should get a native English speaker to proofread the manuscript to correct errors.

Response: Now we have corrected grammatical and spelling error. Previously it was edited and corrected by ‘editage Cactus’-licensed English edition. If again additional English edition needed, they are volunteer to edit again.

Note: Thank you very much for you correct and reasonable comments. We are ready to correct any comments you are giving for us.
