# Supplementary material for: Undernutrition and its determinants among adolescent girls in low land area of Southern Ethiopia
Source: PLoS One. 2021 Jan 12;16(1):e0240677. doi: 10.1371/journal.pone.0240677 (PMC7802945; doi:10.1371/journal.pone.0240677)
Supplement: Attachment — Submitted filename: Response to Reviewers.docx [file pone.0240677.s008.docx]

First of all, we would like to thank the academic editor and Reviewer #2 and Reviewer #3 for their scientific, reasonable, and convincing comments. Really, we have got interesting lesson from them. Thank you very much again!

I tarried to respond to all comments and questions below:

**Response for comments and questions raised by the academic editor**

1. We filled the STROBE CHECKLIST and attached it as a supplementary file
2. We limited the repetition of results at discussion section
3. We ensure that all the reviewers’ comments were well addressed.

Thank you very much for publishing this manuscript. It has taken almost 10 months.

**Response for reviewer#2 comments**

Comment: Variable in Table 3 should read variables

Response: we corrected Variable in Table 3 as variables

**Response for reviewer#3 comments and questions**

1. At the discussion section, the authors should limit the repetition of results

Response: We limited the repetition of results at discussion section

2. The authors should kindly fill the STROBE CHECKLIST and attach it as a supplementary file.

Response: We filled the STROBE CHECKLIST and attached it as a supplementary file

3. Have the authors made all data underlying the findings in their manuscript fully available?

Response: All the data underlying the findings are within the manuscript as well as attached as supplementary file.

Note: Thank you very much for you correct and reasonable comments. We are ready to correct any comments you are giving for us.
